# Direct Cyclization/Chlorination Strategy of Hydrazines for Synthesis of 4-Chloropyrazoles by TCCA

**DOI:** 10.3390/molecules30193841

**Published:** 2025-09-23

**Authors:** Qingfu Deng, Chenglong Ma, Liangzhen Hu, Yan Xiong

**Affiliations:** 1 School of Chemical Engineering, Chongqing Chemical Industry Vocational College, Chongqing 401228, China; 2 School of Chemistry and Chemical Engineering, Chongqing University, Chongqing 401331, China; m15334589338@163.com (C.M.); xiong@cqu.edu.cn (Y.X.)

**Keywords:** cyclization/chlorination, pyrazoles, TCCA

## Abstract

A new method for synthesis of various 4-chloropyrazoles through the direct cyclization/chlorination of hydrazines by using 1,3,5-trichloroisocyanuric acid (TCCA) as both oxidant and chlorinating agent under mild conditions has been proposed. Based on the detailed optimization of the reaction conditions, the substrate generality has been investigated, and the yields of the desired products are up to 92%.

## 1. Introduction

**Figure 1:**
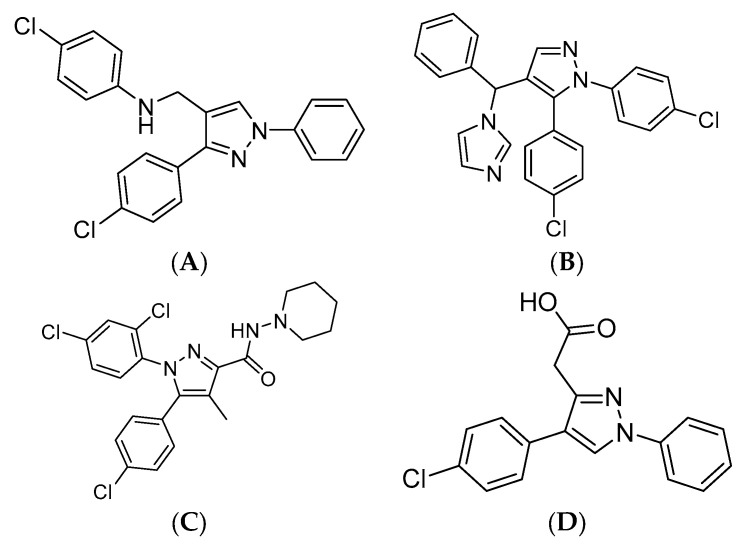
Structures of some pharmacologically important pyrazoles.

Pyrazoles and their derivatives are of particular importance in organic chemistry and pharmaceutical chemistry [1]. As important heterocyclic compounds, pyrazoles exhibit significant biological properties such as antimicrobial [2], anticancer [3,4], analgesic [5], and antihyperglycemic [6] activity. In organic chemistry they can be used as intermediates to synthesize dyes, materials, medicines, etc. [7,8,9,10,11]. Moreover, they also have wide applications in medical treatment [2,12,13,14], agricultural chemicals [15,16], and inhibitors [3,17,18,19]. A great number of pyrazole derivatives have been uncovered with excellent bioactivities, and several classical examples are summarized in Figure 1. 4-chloro-*N*-((3-(4-chlorophenyl)-1-phenyl-1*H*-pyrazol-4-yl)methyl)aniline (Figure 1A) exhibits potent CDK2 inhibitory activities and antiproliferative activities against MCF-7 and B16-F10 cells [3]. Figure 1B has a good antifungal effect on Candida albicans, Cryptococcus neoformans, and Staphylococcus aureus [2]. Rimonabant (Figure 1C) acts as a neurokinin-3 antagonist and a selective cannabinoid CB1 receptor antagonist [20]. Lonazolac (Figure 1D) and its derivatives are important anti-inflammatory agents [12].

Many efforts have been made to find effective ways to integrate the above framework owing to its unique structure. The traditional synthesis of pyrazoles based on the condensation of 1,3-dicarbonyl compounds with hydrazines and the 1,3-dipolar cycloaddition reaction of a dipole with an appropriate dipolarophile is still applicable [21,22]. The cycloadditions of 1,3-dipolar reagents and intramolecular nitrogen addition to alkynes have been developed to obtain pyrazoles [23]. In recent years, the method of synthesizing pyrazole derivatives through metal-catalyzed C–N bond and N–N bond coupling has made great progress [24,25,26,27,28,29]. At the same time, progress has also been made in the synthesis of pyrazole derivatives without metal-mediated molecular oxidation and amination to form C-N bonds [28,30]. However, there are no reports for one-pot construction and fictionalization of pyrazole and the functionalization for the synthesis of pyrazole derivatives. Herein, based on our previous works on the synthesis of nitrogen-containing heterocycles [31,32,33,34], we report a new metal-free synthesis method for substituted pyrazoles, which uses efficient and stable TCCA as both the chlorinating agent and oxidant.

## 2. Results and Discussion

The feasibility of the expected transformation was tested by using 1-phenyl-2-(4-phenylbut-3-en-2-ylidene)hydrazine **1a** as a model substrate and TCCA with lower toxicity as a chlorine source and oxidant in TFE (trifluoroethanol) as the solvent, and the results are summarized in Table 1. To our delight, the conversion of the tested substrate proceeds to the desired 4-chloropyrazole product **3a** with 69% yield at 30 °C for 4 h (entry 1, Table 1). Encouraged by this primary result, the efficiencies of several common solvents were examined, and the result showed that when TFE was used as a solvent, the yield of product **3a** was higher than that of the tested solvents (entries 1–8, Table 1). Next, in the study of temperature effect, there was very little temperature effect in the examined interval (entries 9–11, Table 1). A reduced yield of target product 3a was observed due to excessive or insufficient reaction times (entries 12–13, Table 1). In the end, the effect of the amounts of TCCA and TFE was examined, respectively, and the results indicated that the alteration of these parameters was unfavorable (entries 14–15, Table 1).

In order to further improve the yield of the target compounds, several additives were examined, and the results revealed that the transformation was inhibited by 4Å MS (Entry 1, Table 2). When acid CH_3_COOH was used as an additive, a lower yield of 57% was obtained (Entry 2, Table 2). Then, common organic base pyridine and inorganic base K_2_CO_3_ did not improve the yield of the target product **3a** (entries 3–4, Table 2). Finally, the addition of metal salts did not promote the conversion of the reaction, and the attempt to improve the reactivity of the substrate was unsuccessful (entries 4–7, Table 2).

With the optimal reaction conditions for the cyclization/chlorination of the standard substrate in hand, the substrate scope was then explored, and the results were summarized in Table 3. Various substrates with diverse substitutions on group R^1^ in phenyl and R^2^ linked to the imine carbon were studied. The results showed that when the substituent R^2^ is H, various results were produced due to different substitutions of R^1^. For example, when R^1^ is H, the formation of target product **3b** is not observed, and only the cyclized product **3b′** is obtained. On the contrary, when the substituent R^1^ is an electron-donating group, such as methoxy at the *para*-position of the phenyl, a lower yield of **3c′** can be obtained. At the same time, it was also found that when R^2^ was set to methyl, the desired products **3d**–**3i** could be obtained in moderate to good yields (40–92%) regardless of whether the phenyl of cinamaldehyde was substituted by a *para*-electron-withdrawing or *para*-electron-donating substituent. In addition, when **1j** with double methoxy on the phenyl of cinamaldehyde was used as substrate, the target product **3j** could also be obtained, although the yield was not quite high. Substrates with R^2^ set to be phenyl or p-methylphenyl were also applied to the reaction, and the target products **3l**–**3p** were obtained regardless of whether R^1^ was *para*-substituted on the phenyl by an electron-withdrawing or electron-donating group. However, the yields were relatively low, and it may be due to the large steric hindrance caused by the introduction of phenyl or p-methylphenyl, and these results indicate that the steric hindrance effect of the R^2^ group has a great effect on the transformation (**3d** vs. **3k**, **3e** vs. **3l**, **3g** vs. **3m**, **3h** vs. **3n**).

**Scheme 1:**
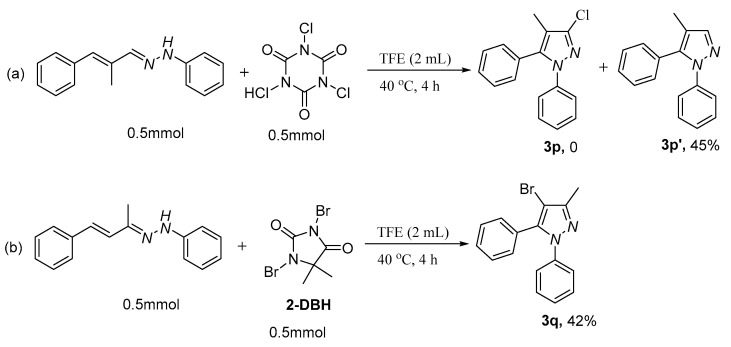
Further exploration with substrates.

Furthermore, in order to obtain more pyrazole derivatives, (*E*)-1-((*E*)-2-methyl-3-phenylallylidene)-2-phenylhydrazine was employed as a substrate to form the corresponding chloropyrazole, while the expected chlorinated product **3p** was not detected, and the cyclization product 3p’ was obtained in a 45% yield (Scheme 1a). Meanwhile, bromocyclization of **1a** using 2-DBH as a replacement for TCCA afforded brominated pyrazole **3q** (Scheme 1b).

Following the synthesis of 4-bromopyrazole **3q**, Suzuki–Miyaura cross-coupling reactions were investigated for aryl functionalization (Table 4). Coupling **3q** with phenylboronic acid proceeded efficiently, utilizing the bromine atom as a more reactive leaving group to afford target product **4a** in 45% yield. Reactions with methylphenylboronic acid, methoxyphenylboronic acid, biphenylboronic acid, and chlorophenylboronic acid afforded products in comparable yields under varying electronic effects (electron-donating or -withdrawing). Notably, coupling with 4-(trimethylsilyl)phenylboronic acid achieved an unexpectedly high yield of 86% (**4d**). This exceptional result may be attributed to weak coordination between the trimethylsilyl group and palladium, potentially facilitating a directing effect. Conversely, attempts to couple **3q** with cyclohexylboronic acid failed to generate **4g**, as cyclohexyl lacks aromatic electronic effects and conjugation capabilities. Similarly, no products **4h** or **4i** were observed with aliphatic or heterocyclic boronic acids.

**Scheme 2:**
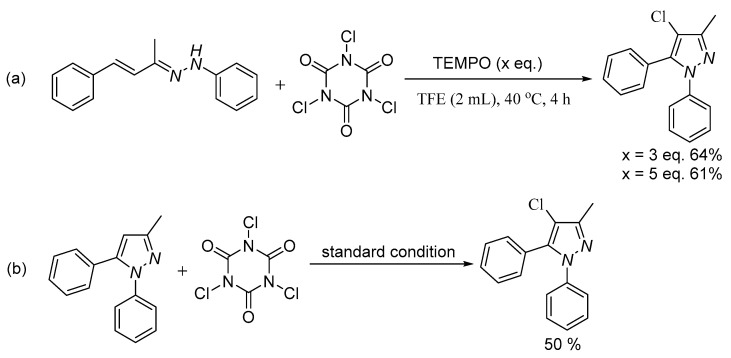
Control experiments.

To clarify the mechanism, controlled experiments were conducted. To the reaction mixture, 3 to 5 equiv. of radical scavenger 2,2,6,6-tetramethylpiperidinooxy (TEMPO) was added; however, the desired product was still obtained with isolated yields of 64% and 61%, respectively (Scheme 2a). These results ruled out the possibility of a radical pathway. Then, the chlorination of the pyrazole was examined, and the 3-methyl-1,5-diphenyl-1*H*-pyrazole could react under the standard conditions and provide the corresponding chlorinated product in moderate yield (Scheme 2b). This reveals that the chlorination process can occur after the cyclization.

**Figure 2:**
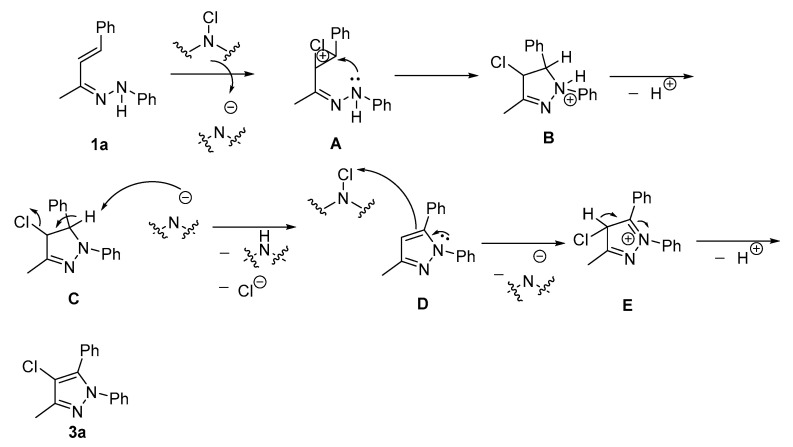
Possible mechanism.

Based on the experimental results and literature precedents [35], a plausible reaction mechanism is proposed (Figure 2). Initially, the chloramine moiety of oxidant TCCA reacts with hydrazone **1a**, generating intermediate **A** alongside an amide anion. Intermediate **A** undergoes intramolecular cyclization to form **B**. Subsequent deprotonation of this intermediate yields **C**. Under the influence of the amide anion, **C** undergoes dechlorination (elimination of Cl^−^) to form a double bond, affording **D**. **D** is further oxidized by TCCA to cationic intermediate **E**, which finally undergoes deprotonation to afford the target product **3a**.

## 3. Conclusions

In summary, we have developed a novel and straightforward method for the chlorination/cyclization of hydrazine substrates promoted by TCCA (trichloroisocyanuric acid). Under optimized conditions, a series of 4-chloropyrazole derivatives was synthesized via the one-pot sequential construction of an intramolecular C–N bond and construction of a C–Cl bond. TCCA serves as a highly efficient, low-cost, and low-toxicity oxidant and chlorine source, which makes this new method both economical and environmentally benign. This one-pot strategy enables concurrent construction of the pyrazole ring and its functionalization. The method is applicable to the preparation of diverse novel chloropyrazole derivatives, which are expected to play significant roles in pharmaceutical and agrochemical research.

## 4. Experimental

### 4.1. General Information

CDCl_3_ was used as the solvent to measure its ^1^H and ^13^C spectra with a 400/100 MHz NMR or 500/125 MHz NMR Bruker Avance spectrometer from Chongqing, China at 20–25 °C. Tetramethylsilane (TMS, δ = 0.00 ppm) played the role of an internal standard to report chemical shifts in parts per million. The chemical reagents involved in the experiment can be purchased directly from merchants and are all analytically pure. All the weighing processes were carried out at room temperature in air, and all reactions were carried out under normal pressure unless otherwise specified

### 4.2. General Procedure for the Synthesis of Pyrazole Derivatives (**3**)

The oxidant TCCA (0.5 mmol, 1.0 equiv.) was added to the stirring solution of hydrazine substrate **1** (0.5 mmol) in TFE (2 mL), and then the mixture was reacted for 4 h at 40 °C. After the reaction, it was cooled to room temperature and quenched with a saturated solution of Na_2_S_2_O_3_ (1–2 mL), diluted with EtOAc (5 mL), and extracted with ethyl acetate (3 × 15 mL). The separated organic solution was dried with Mg_2_SO_4_, and the solvent was evaporated in vacuo. The resulting residue was purified by column chromatography on a silica gel column using EtOAc-petroleum ether (1:150) as an eluent to obtain target products.

### 4.3. 4-Chloro-3-methyl-1,5-diphenyl-1H-pyrazole (**3a**)

1-Phenyl-2-(4-phenylbut-3-en-2-ylidene)hydrazine **1a** (0.5 mmol, 118 mg) and TCCA (0.5 mmol, 116 mg) were employed to afford 100.8 mg (75%) of the indicated product as a yellow oil (R_f_ = in 4:1 petroleum ether/ethyl acetate); ^1^H NMR (500 MHz, CDCl_3_) δ 7.33 (m, 3H), 7.31–7.25 (m, 5H), 7.23–7.20 (m, 2H), 2.39 (s, 3H); ^13^C NMR (100 MHz, CDCl_3_) δ 147.1, 140.0, 139.1, 129.9, 129.1, 128.9, 128.74, 128.65, 127.5, 124.9, 110.5, 11.7.

### 4.4. General Procedure for the Synthesis of Arylated Pyrazole Derivatives (**4**)

To a solution of **3q** (0.3 mmol), K_2_CO_3_ (0.72 mmol), and R-B(OH)_2_ (0.36 mmol) in toluene/water (6:1, *v*/*v*) mixture (3 mL), Pd(PPh_3_)_4_ (0.015 mmol) was added under air. After stirring for 10 h at 100 °C, the solvent was removed by vacuum, and the resulting residue was purified by column chromatography on a silica gel column using EtOAc-petroleum ether (1:150) as an eluent to obtain target products.

General information, experimental details, characterization data, and ^1^H and ^13^C NMR spectra for all the synthesized compounds are available as Supplementary Information. This material can be found via the “Supplementary Materials (^1^H and ^13^C NMR spectra for **3** and **4**)” section of this article’s webpage.

## Figures and Tables

**Table 1:** Optimizations of reaction conditions ^a^.

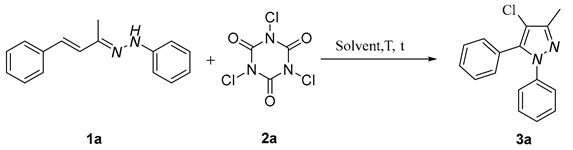					
Entry	Solvent (mL)	t/h	T/°C	TCCA (eq.)	Yield ^b^ (%)
1	TFE	4	30	1.0	69
2	MeOH	4	30	1.0	8
3	EtOH	4	30	1.0	38
4	HFIP	4	30	1.0	40
5	DMF	4	30	1.0	32
6	HOAc	4	30	1.0	25
7	1,4-Dioxane	4	30	1.0	14
8	DCM	4	30	1.0	6
9	TFE	4	0	1.0	64
10	TFE	4	40	1.0	75, (65) ^c^
11	TFE	4	60	1.0	70
12	TFE	2	40	1.0	70
13	TFE	6	40	1.0	68
14	TFE	4	40	0.8	68
15	TFE	4	40	1.2	58

^a^ reaction conditions: **1a** (0.5 mmol), **2a** (0.5 mmol) in solvent (2 mL) at T/°C for t h. ^b^ determined after isolation. ^c^ 5 mL of TFE.

**Table 2:** Test of additives ^a^.

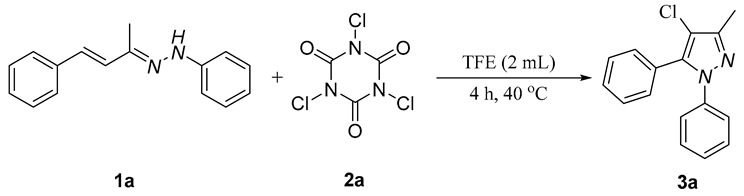		
Entry	Additive (5 wt%)	Yield ^b^ (%)
1	4 Å MS	47
2	CH_3_COOH	57
3	Pyridine	63
4	K_2_CO_3_	49
5	Zn(OAc)_2_·2H_2_O	63
6	Cu(OAc)_2_·2H_2_O	64
7	Fe(OAc)_2_	72

^a^ reaction conditions: **1a** (0.5 mmol), **2a** (0.5 mmol), and additive (5 wt%) in TFE (2 mL) at 40 °C for 4 h. ^b^ determined after isolation.

**Table 3:** Investigation of substrates ^a^.

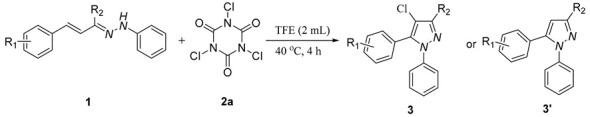			
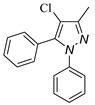	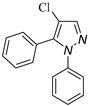	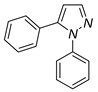	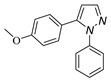
**3a**, 75% ^b^	**3b**, 0	**3b′**, 25%	**3c′**, 45%
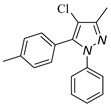	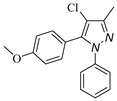	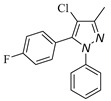	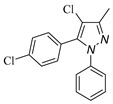
**3d**, 59%	**3e**, 40%	**3f**, 40%	**3g**, 43%
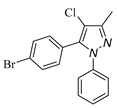	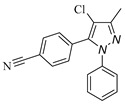	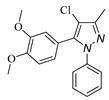	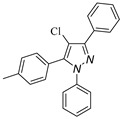
**3h**, 45%	**3i**, 92%	**3j**, 72%	**3k**, 15%
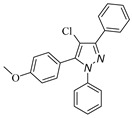	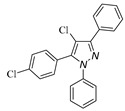	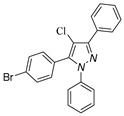	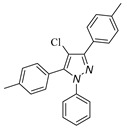
**3l**, 18%	**3m**, 18%	**3n**, 25%	**3o**, 15%

^a^ reaction conditions: **1a** (0.5 mmol), **2a** (0.5 mmol) in TFE (2 mL) at 40 °C for 4 h. ^b^ determined after isolation.

**Table 4:** Arylation scope of 4-Bromopyrazole ^a^.

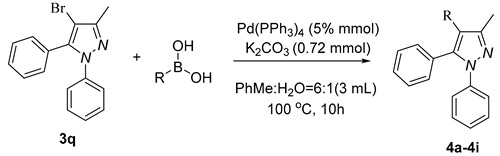		
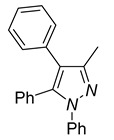	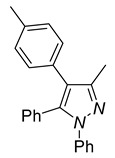	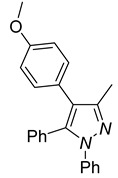
**4a**, 45% ^b^	**4b**, 48%	**4c**, 40%
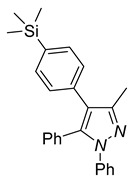	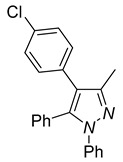	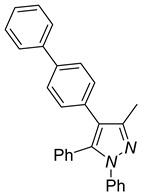
**4d**, 86%	**4e**, 37%	**4f**, 58%
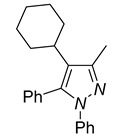	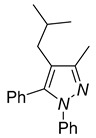	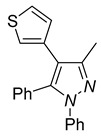
**4g**, 0%	**4h**, 0%	**4i**, 0%

^a^ Reaction conditions: **3q** (0.3 mmol), R-B(OH)_2_ (0.36 mmol). ^b^ Determined after isolation.

## Data Availability

The original contributions presented in this study are included in the article/Supplementary Material. Further inquiries can be directed to the corresponding authors.

## Supplementary Materials

The following supporting information can be downloaded at: https://www.mdpi.com/article/10.3390/molecules30193841/s1. References [29,36,37] are cited in the Supplementary Materials.
