# Supplementary material for: Direct Cyclization/Chlorination Strategy of Hydrazines for Synthesis of 4-Chloropyrazoles by TCCA
Source: Molecules. 2025 Sep 23;30(19):3841. doi: 10.3390/molecules30193841 (PMC12525685; doi:10.3390/molecules30193841)
Supplement: Supplementary file 1 [file molecules-30-03841-s001.zip › molecules-3851335-supplementary.pdf]

Direct Cyclization/Chlorination Strategy of Hydrazines for Synthesis of 4-Chloropyrazoles by TCCA

Qingfu Deng [1,*], Chenglong Ma [2], Liangzhen Hu [2,*] and Yan Xiong [2]

[1] School of Chemical Engineering, Chongqing Chemical Industry Vocational College,
Chongqing 401228, China
[2] School of Chemistry and Chemical Engineering, Chongqing University, Chongqing 401331, China; m15334589338@163.com (C.M.); xiong@cqu.edu.cn (Y.X.)
* Correspondence: 15320254920@163.com (Q.D.); huliangzhen@cqu.edu.cn (L.H.)

Tables of content

Experimental Section …………………………………………………………… S2

General information …………………………………………………………… S2

Synthesis of 3 ……………………………………………………………………S2

Copies of ^{1}H and ^{13}C NMR Spectra ……………………………………………S8

References ……………………………………………………………………… S36

Experimental Section

General information. $CDCl_3$ was acted as the solvent to measure the product its ^1H and ^{13}C spectra with 400/100MHz NMR or 500/125MHz NMR spectrometer at 20-25°C. Tetramethylsilane (TMS, δ = 0.00 ppm) was played the role of an internal standard to report the product its ^1H and ^{13}C in parts per million. The chemical reagents involved in the experiment can be purchased directly from merchants and are all analytically pure. All weighing processes are carried out in room temperature in air and all reactions are carried out under normal pressure unless otherwise specified

General procedure for the synthesis of pyrazole derivatives(**3**)

The oxidant TCCA (0.5 mmol, 1.0 equiv.) was added to the stirring solution of hydrazine substrate **1** (0.5 mmol), in TFE (2 mL) then the mixture was reacted for 4 hours at 40 °C. After the reaction, it was cooled to room temperature and quenched with saturated solution of $Na_2S_2O_3$ (1-2 mL), diluted with EtOAc (5 mL), and extracted with ethyl acetate (3×15 mL). The separated organic solution was dried with Mg_2SO_4 and the solvent was evaporated in vacuo. The resulting residue was purified by column chromatography on silica gel column by using EtOAc-petroleum ether (1:150) as eluent to obtain target products.

4-chloro-3-methyl-1,5-diphenyl-1H-pyrazole (**3a**)

1-phenyl-2-(4-phenylbut-3-en-2-ylidene)hydrazine **1a** (0.5 mmol, 118 mg) and TCCA (0.5 mmol, 116 mg) were employed to afford 100.8 mg (75%) of the indicated product as a yellow oil (R_f = 0.50 in 1:20 petroleum ether/ethyl acetate); ^1H NMR (500 MHz, $CDCl_3$) δ 7.33-7.33 (m, 3H), 7.31-7.25 (m, 5H), 7.23-7.20 (m, 2H), 2.39 (s, 3H); ^{13}C NMR (100 MHz, $CDCl_3$) δ 147.1, 140.0, 139.1, 129.9, 129.1, 128.9, 128.74, 128.65, 127.5, 124.9, 110.5, 11.7.

1,5-diphenyl-1H-pyrazole (**3b′**)

1-phenyl-2-(3-phenylallylidene)hydrazine **1b** (0.5 mmol, 111 mg) and TCCA (0.5 mmol, 116 mg) were employed to afford 27.8 mg (25%) of the indicated product as a yellow oil (R_f= 0.16 in 1:20 petroleum ether/ethyl acetate); ^1H NMR (500 MHz, $CDCl_3$) δ 7.72 (s, 1H), 7.34-7.29 (m, 5H), 7.26-7.23 (m, 4H), 6.51 (s, 1H); ^{13}C NMR (125 MHz,

$CDCl_3$) δ 143.2, 140.8, 138.8, 133.2, 130.5, 129.2, 129.0, 128.8, 128.6, 126.4, 108.4. The spectral data were in agreement with those reported previously for this compound.[1]

5-(4-methoxyphenyl)-1-phenyl-1H-pyrazole (**3c′**)

1-(3-(4-methoxyphenyl)allylidene)-2-phenylhydrazine **1c** (0.5 mmol, 126 mg) and TCCA (0.5 mmol, 116 mg) were employed to afford 64.1 mg (45%) of the indicated product as a yellow oil (R_f = 0.30 in 1:20 petroleum ether/ethyl acetate); ^{1}H NMR (500 MHz, $CDCl_3$) δ 7.62 (s, 1H), 7.15 (m, 5H), 7.07 (d, J = 7.5 Hz, 2H), 6.78 (d, J = 8.0 Hz, 2H), 6.37 (s, 1H), 3.74 (s, 3H); ^{13}C NMR (125 MHz, $CDCl_3$) δ 159.9, 143.1, 140.7, 138.9, 133.1, 130.2, 129.2, 126.4, 122.8, 114.2, 107.9, 55.5. The spectral data were in agreement with those reported previously for this compound.[2]

*4-chloro-3-methyl-1-phenyl-5-(p-tolyl)-1H-pyrazole (***3d***)*

1-phenyl-2-(4-(p-tolyl)but-3-en-2-ylidene)hydrazine **1d** (0.5 mmol, 125 mg) and TCCA (0.5 mmol, 116 mg) were employed to afford 82.8 mg (59%) of the indicated product as a yellow oil (R_f = 0.59 in 1:20 petroleum ether/ethyl acetate); ^{1}H NMR (500 MHz, $CDCl_3$) δ 7.29-7.20 (m, 5H), 7.17-7.13 (m, 4H), 2.37 (s, 3H), 2.34 (s, 3H); ^{13}C NMR (125 MHz, $CDCl_3$) δ 147.0, 140.1, 139.1, 138.9, 129.6, 129.4, 129.0, 127.3, 125.7, 124.8, 110.2, 21.5, 11.7.

4-chloro-5-(4-methoxyphenyl)-3-methyl-1-phenyl-1H-pyrazole (**3e**)

1-(4-(4-methoxyphenyl)but-3-en-2-ylidene)-2-phenylhydrazine **1d** (0.5 mmol, 133 mg) and TCCA (0.5 mmol, 116 mg) were employed to afford 59.7 mg (40%) of the indicated product as a yellow oil (R_f = 0.35 in 1:20 petroleum ether/ethyl acetate); ^{1}H NMR (500 MHz, $CDCl_3$) δ 7.31-7.18 (m, 7H), 6.87 (d, J = 7.5 Hz, 2H), 3.80 (s, 3H), 2.37 (s, 3H); ^{13}C NMR (125 MHz, $CDCl_3$) δ 159.9, 147.0, 140.1, 138.9, 131.1, 129.0, 127.3, 124.8, 120.9, 114.1, 110.1, 55.4, 11.7.

4-chloro-5-(4-fluorophenyl)-3-methyl-1-phenyl-1H-pyrazole (**3f**)

1-(4-(4-fluorophenyl)but-3-en-2-ylidene)-2-phenylhydrazine (**1f**) (0.5 mmol, 127 mg) and TCCA (0.5 mmol, 116 mg) were employed to afford 57.3 mg (40%) of the indicated product as a yellow oil (R_f = 0.40 in 1:20 petroleum ether/ethyl acetate); ^{1}H NMR (400 MHz, $CDCl_3$) δ 7.24-7.10 (m, 7H), 6.98-6.93 (m, 2H), 2.29 (s, 3H); ^{13}C NMR (125 MHz,$CDCl_3$) δ 162.9 (d, J = 247.25), 147.1, 139.9, 138.1, 131.7 (d, J = 8.75), 129.1, 127.6, 124.9, 124.7 (d, J = 2.5), 115.9 (d, J = 21.25), 110.5, 11.7.

4-chloro-5-(4-chlorophenyl)-3-methyl-1-phenyl-1H-pyrazole (**3g**)

1-(4-(4-chlorophenyl)but-3-en-2-ylidene)-2-phenylhydrazine (**1g**) (0.5 mmol, 135 mg) and TCCA (0.5 mmol, 116 mg) were employed to afford 65.2 mg (43%) of the indicated product as a yellowish oil (R_f = 0.54 in 1:20 petroleum ether/ethyl acetate); ^{1}H NMR (400 MHz, $CDCl_3$) δ 7.24-7.15 (m, 5H), 7.13-7.09 (m, 4H), 2.29 (s, 3H); ^{13}C NMR (100 MHz, $CDCl_3$) δ 147.2, 139.7, 137.8, 135.0, 131.1, 129.2, 129.0, 127.7, 127.1, 124.9, 110.6, 11.6.

5-(4-bromophenyl)-4-chloro-3-methyl-1-phenyl-1H-pyrazole (**3h**)

1-(4-(4-bromophenyl)but-3-en-2-ylidene)-2-phenylhydrazine (**1h**) (0.5 mmol, 135 mg) and TCCA (0.5 mmol, 116 mg) were employed to afford 78.2 mg (45%) of the indicated product as a white solid (R_f = 0.51 in 1:20 petroleum ether/ethyl acetate; mp 102.5-104 °C); ^{1}H NMR (400 MHz, $CDCl_3$) δ 7.39 (d, J = 10.5 Hz, 2H),7.25-7.17 (m, 3H), 7.13-7.10 (m, 2H), 7.07-7.04 (m, 2H), 2.29 (s, 3H); ^{13}C NMR (100 MHz, $CDCl_3$) δ 147.2, 139.7, 137.8, 131.9, 131.3, 129.2, 127. 127.5, 124, 123.3, 110.6, 11.7.

4-(4-chloro-3-methyl-1-phenyl-1H-pyrazol-5-yl)benzonitrile (**3i**)

4-(3-(2-phenylhydrazono)but-1-en-1-yl)benzonitrile (**1i**) (0.5 mmol, 131 mg) and TCCA (0.5 mmol, 116 mg) were employed to afford 135.1 mg (92%) of the indicated product as a white solid (R_f = 0.34 in 1:20 petroleum ether/ethyl acetate; mp 88-89.6 °C); ^{1}H NMR (400 MHz, $CDCl_3$) δ 7.65-7.62 (m, 2H), 7.40-7.38 (m, 2H), 7.36-7.30 (m, 3H), 7.19-7.15 (m, 2H), 2.38 (s, 3H); ^{13}C NMR (100 MHz, $CDCl_3$) δ 147.6, 139.5, 137.0, 133.2, 132.4, 130.3, 129.4, 128.1, 125.0, 118.4, 112.5, 111.3, 11.6.

4-chloro-5-(3,4-dimethoxyphenyl)-3-methyl-1-phenyl-1H-pyrazole (**3j**)

1-(4-(3,4-dimethoxyphenyl)but-3-en-2-ylidene)-2-phenylhydrazine (**1j**) (0.5 mmol, 148 mg) and TCCA (0.5 mmol, 116 mg) were employed to afford 118.8 mg (72%) of the indicated product as a yellow solid (R_f = 0.29 in 1:20 petroleum ether/ethyl acetate; mp 86.1-87.5 °C); ^{1}H NMR (500 MHz, $CDCl_3$) δ 7.23-7.13 (m, 5H), 6.78 (dd, J = 28.0, 8.0Hz, 2H), 6.61 (s, 1H), 3.79(s, 3H), 3.57 (s, 3H), 2.29 (s, 3H); ^{13}C NMR (125 MHz, $CDCl_3$) δ 149.4, 148.8, 146.9, 140.1, 138.9, 129.0, 127.4, 124.9, 122.6, 120.9, 112.8, 111.1, 110.0, 55.93, 55.85, 11.7.

4-chloro-1,3-diphenyl-5-(p-tolyl)-1H-pyrazole (**3k**)

1-phenyl-2-(1-phenyl-3-(p-tolyl)allylidene)hydrazine (**1k**) (0.5 mmol, 156 mg) and TCCA (0.5 mmol, 116 mg) were employed to afford 25.9 mg (15%) of the indicated product as a yellow solid (R_f = 0.46 in 1:20 petroleum ether/ethyl acetate; mp 134.2-136.5 °C); ^{1}H NMR (500 MHz, $CDCl_3$) δ 8.02 (d, J = 8.0Hz, 2H), 7.48-7.38 (m, 5H),

7.35-7.29 (m, 2H), 7.21 (d, J = 8.0Hz, 2H), 7.12 (d, J = 8.0Hz, 2H), 2.33 (s, 3H); ^{13}C NMR (125 MHz, $CDCl_3$) δ 148.7, 142.4, 139.2, 137.9, 132.5, 131.7, 130.55, 130.53, 130.2, 129.5, 129.3, 128.6, 127.9, 127.6, 125.0, 107.8, 21.6.

4-chloro-5-(4-methoxyphenyl)-1,3-diphenyl-1H-pyrazole (**3l**)

1-(3-(4-methoxyphenyl)-1-phenylallylidene)-2-phenylhydrazine **1l** (0.5 mmol, 164 mg) and TCCA (0.5 mmol, 116 mg) were employed to afford 32.5 mg (18%) of the indicated product as a white solid (R_f = 0.37 in 1:20 petroleum ether/ethyl acetate; mp 138.8-140.1 ºC); ^{1}H NMR (500 MHz, $CDCl_3$) δ 8.03-8.00 (m, 2H), 7.49-7.38 (m, 5H), 7.37-7.29 (m, 2H), 7.27-7.23 (m, 2H), 6.88-6.82 (m, 2H), 3.79 (s, 3H); ^{13}C NMR (125 MHz, $CDCl_3$) δ 160.0, 148.6, 142.2, 137.9, 132.5, 131.7, 131.0, 130.6, 130.5, 130.2, 128.6, 127.9, 127.7, 120.2, 114.0, 107.6, 55.4.

4-chloro-5-(4-chlorophenyl)-1,3-diphenyl-1H-pyrazole (**3m**)

1-(3-(4-chlorophenyl)-1-phenylallylidene)-2-phenylhydrazine **1m** (0.5 mmol, 166 mg) and TCCA (0.5 mmol, 116 mg) were employed to afford 32.9 mg (18%) of the indicated product as a yellowish solid (R_f = 0.46 in 1:20 petroleum ether/ethyl acetate; mp 136.7-138.2 ºC); ^{1}H NMR (400 MHz, $CDCl_3$) δ 8.02-7.99 (m, 2H), 7.49-7.39 (m, 5H), 7.39-7.33 (m, 3H), 7.31-7.26 (m, 3H), 7.26-7.24 (m, 1H); ^{13}C NMR (100 MHz, $CDCl_3$) δ 147.0, 139.9, 139.0, 129.8, 129.1, 129.0, 128.8, 128.62, 128.57, 127.4, 124.8, 110.4, 11.6.

5-(4-bromophenyl)-4-chloro-1,3-diphenyl-1H-pyrazole (**3n**)

1-(3-(4-bromophenyl)-1-phenylallylidene)-2-phenylhydrazine **1n** (0.5 mmol, 188 mg) and TCCA (0.5 mmol, 116 mg) were employed to afford 51.2 mg (25%) of the indicated product as a white solid (R_f = 0.47 in 1:20 petroleum ether/ethyl acetate; mp 152.2-154.1 ºC); ^{1}H NMR (400 MHz, $CDCl_3$) δ 8.02-7.99 (m, 2H), 7.49-7.32 (m, 10H), 7.19 (d, J = 10.5Hz, 2H); ^{13}C NMR (100 MHz, $CDCl_3$) δ 148.9, 141.2, 137.5, 132.3, 131.9, 131.4, 131.1, 130.8, 130.1, 128.8, 128.6, 127.9, 127.8, 126.8, 123.6, 108.1.

4-chloro-1-phenyl-3,5-di-p-tolyl-1H-pyrazole (**3o**)

1-(1,3-di-p-tolylallylidene)-2-phenylhydrazine **1o** (0.5 mmol, 163 mg) and TCCA (0.5 mmol, 116 mg) were employed to afford 26.9 mg (15%) of the indicated product as a white solid (R_f = 0.46 in 1:20 petroleum ether/ethyl acetate; mp 173-174.3 ºC); ^{1}H NMR (400 MHz, $CDCl_3$) δ 7.91 (d, *J* = 8.4Hz, 2H), 7.44-7.37 (m, 2H), 7.34-7.23 (m, 5H), 7.20 (d, *J* = 8.0Hz, 2H), 7.11 (d, *J* = 8.0Hz, 2H); ^{13}C NMR (100 MHz, $CDCl_3$) δ

148.7, 142.3, 139.1, 138.4, 137.9, 130.50, 130.45, 130.2, 129.4, 129.3, 129.2, 128.8, 127.8, 127.6, 125.0, 107.6, 21.5.

3-chloro-4-methyl-1,5-diphenyl-1H-pyrazole (**3p'**)

1-(2-methyl-3-phenylallylidene)-2-phenylhydrazine **1p** (0.5 mmol, 118 mg) and TCCA (0.5 mmol, 116 mg) were employed to afford 60.5 mg (45%) of the indicated product as a yellowish oil (R_f = 0.19 in 1:20 petroleum ether/ethyl acetate); ^{1}H NMR (500 MHz, $CDCl_3$) δ 7.60 (s, 1H), 7.33-7.31 (m, 3H), 7.27-7.16 (m, 7H), 2.11 (s, 3H); ^{13}C NMR (125 MHz, $CDCl_3$) δ 141.3, 140.5, 140.0, 130.8, 130.0, 128.8, 128.6, 128.2, 126.9, 124.8, 116.5, 9.4. The spectral data were in agreement with those reported previously for this compound.[3]

3-methyl-1,4,5-triphenyl-1H-pyrazole (**4a**)

The crude product was purified by column chromatography (petroleum ether/ethyl acetate = 40/1, v/v). Yield: 41.9 mg, 0.13 mmol, 45 %, white solid, mp = 171−173°C; ^{1}H NMR (400 MHz, $CDCl_3$) δ 7.29-7.28 (m, 4H), 7.26-7.16 (m, 7H), 7.15-7.13 (m, 2H), 7.05-7.03 (m, 2H), 2.40 (s, 3H); ^{13}C NMR (100 MHz, $CDCl_3$) δ 148.0, 140.5, 140.2, 133.4, 130.5, 130.1, 128.9, 128.5, 128.4, 128.2, 127.1, 126.5, 125.3, 121.6, 12.9.

3-methyl-1,5-diphenyl-4-(p-tolyl)-1H-pyrazole (**4b**)

The crude product was purified by column chromatography (petroleum ether/ethyl acetate = 40/1, v/v). Yield: 46.7 mg, 0.14 mmol, 48 %, white solid, mp = 158−160°C; ^{1}H NMR (400 MHz, $CDCl_3$) δ 7.31-7.17 (m, 9H), 7.10-7.02 (m, 5H), 2.39 (s, 3H), 2.33 (s, 3H); ^{13}C NMR (100 MHz, $CDCl_3$) δ 148.1, 140.3, 140.2, 136.1, 130.6, 130.5, 130.4, 130.0, 129.1, 128.9, 128.5, 128.2, 127.0, 125.3, 121.5, 21.4, 13.0.

4-(4-methoxyphenyl)-3-methyl-1,5-diphenyl-1H-pyrazole (**4c**)

The crude product was purified by column chromatography (petroleum ether/ethyl acetate = 40/1, v/v). Yield: 40.9 mg, 0.12 mmol, 40 %, white solid, mp = 130−132°C; ^{1}H NMR (400 MHz, $CDCl_3$) δ 7.23-7.10 (m, 8H), 6.98 (t, J = 8.4Hz, 4H), 7.75 (d, J = 8.4Hz, 2H), 3.72 (S, 3H), 2.31 (s, 3H); ^{13}C NMR (100 MHz, $CDCl_3$) δ 158.3, 148.1, 140.2, 131.2, 130.6, 130.5, 128.9, 128.5, 128.1, 127.0, 125.2, 121.3, 113.9, 55.3, 12.9.

3-methyl-1,5-diphenyl-4-(4-(trimethylsilyl)phenyl)-1H-pyrazole (**4d**)

The crude product was purified by column chromatography (petroleum ether/ethyl acetate = 40/1, v/v). Yield: 97.1 mg, 0.25 mmol, 85 %, white solid, mp = 106−108°C; ^{1}H NMR (400 MHz, $CDCl_3$) δ 7.42 (d, J = 8.0Hz, 3H), 7.29-7.27 (m, 1H), 7.26-7.19 (m, 7H), 7.12 (d, J = 8.0Hz, 2H), 7.07-7.55 (m, 2H), 2.42 (s, 3H), 0.26 (s, 9H); ^{13}C

NMR (100 MHz, $CDCl_3$) δ 1448.1, 140.5, 140.1, 138.2, 133.7, 133.4, 130.5, 129.3, 128.9, 128.5, 128.2, 127.1, 125.3, 121.5, 107.9, 13.1, 1.21.

4-(4-chlorophenyl)-3-methyl-1,5-diphenyl-1H-pyrazole (**4e**)

The crude product was purified by column chromatography (petroleum ether/ethyl acetate = 40/1, v/v). Yield: 38.0 mg, 0.11 mmol, 37 %, white solid, mp = 181−182°C; ^{1}H NMR (400 MHz, $CDCl_3$) δ 7.31-7.28 (m, 3H), 7.25-7.19 (m, 7H), 7.07-7.01 (m, 4H), 2.39 (s, 3H); ^{13}C NMR (100 MHz, $CDCl_3$) δ 147.8, 140.6, 140.0, 132.5, 132.0, 131.3, 130.4, 130.2, 129.0, 128.66, 128.64, 128.4, 127.2, 125.3, 120.4, 12.9.

4-([1,1'-biphenyl]-4-yl)-3-methyl-1,5-diphenyl-1H-pyrazole (**4f**)

The crude product was purified by column chromatography (petroleum ether/ethyl acetate = 40/1, v/v). Yield: 67.2 mg, 0.17 mmol, 58 %, white solid, mp = 159 −161°C; ^{1}H NMR (400 MHz, $CDCl_3$) δ 7.60 (d, J = 7.6Hz, 2H), 7.52 (d, J = 8.0Hz, 2H), 7.43 (t, J = 7.6Hz, 2H), 7.35-7.26 (m, 5H), 7.26-7.20 (m, 6H), 7.09 (m, 2H), 2.45 (s, 3H); ^{13}C NMR (100 MHz, $CDCl_3$) δ 148.1, 140.9, 140.5, 140.2, 139.1, 132.5, 130.5, 130.4, 128.9, 128.9, 128.6, 128.3, 127.4, 127.10, 127.05, 125.3, 121.1, 13.1.

Copies of ^{1}H and ^{13}C NMR Spectra

4-chloro-3-methyl-1,5-diphenyl-1H-pyrazole (**3a**)

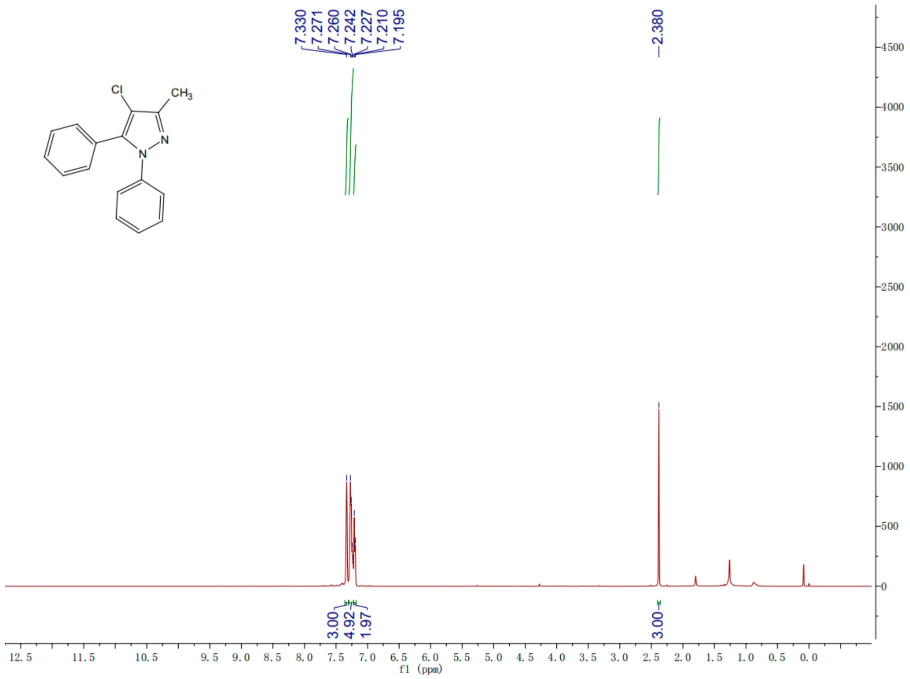

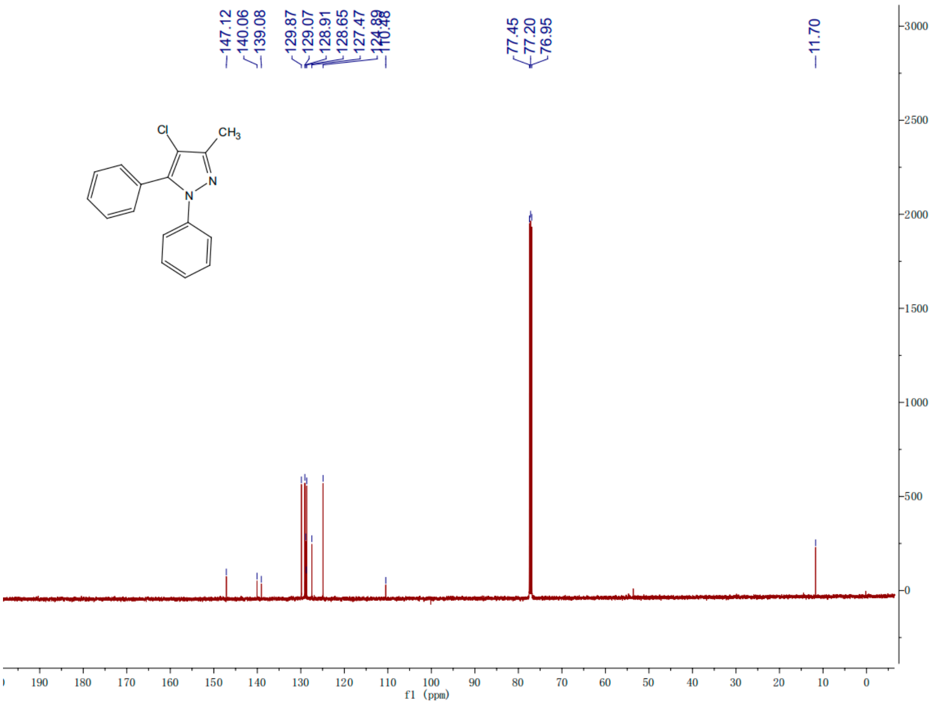

1,5-diphenyl-1H-pyrazole (**3b′**)

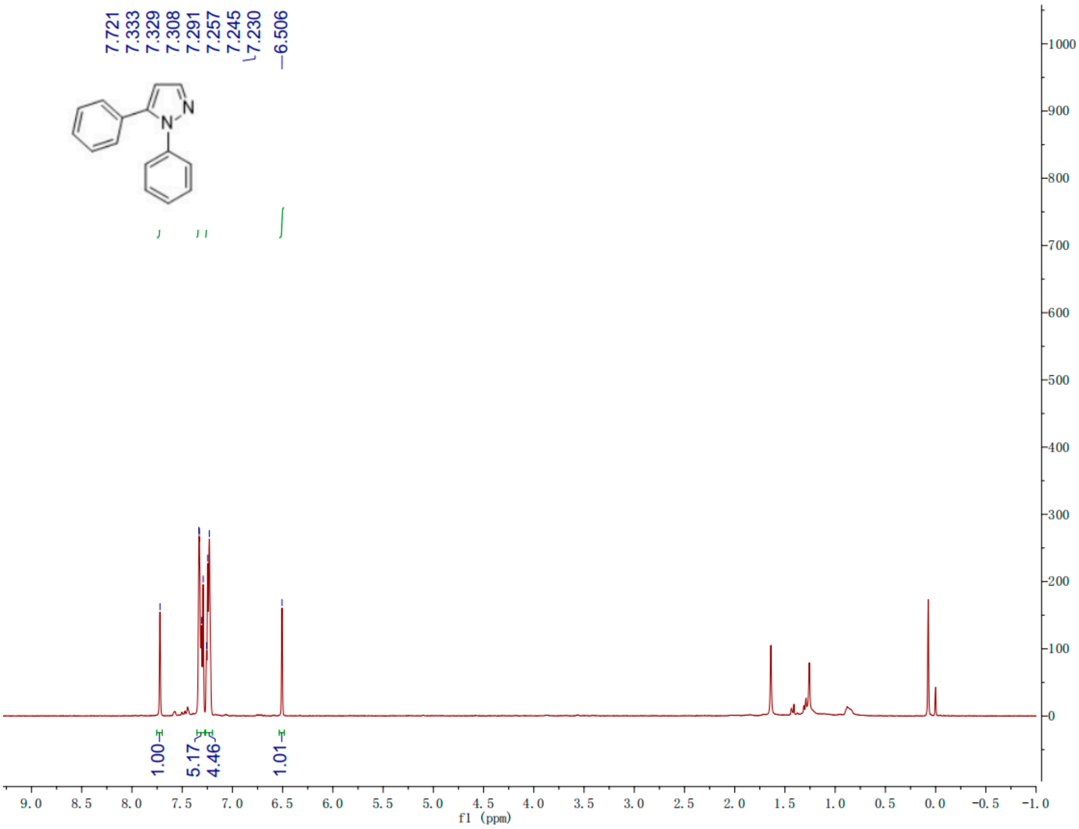

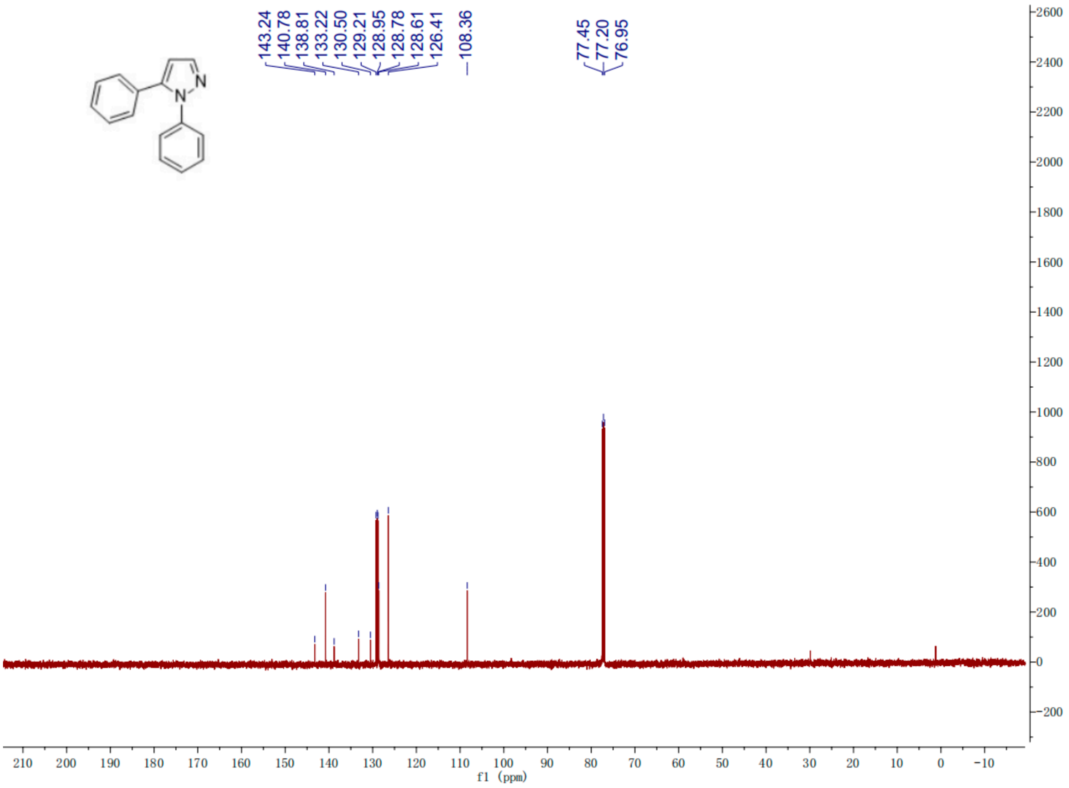

5-(4-methoxyphenyl)-1-phenyl-1H-pyrazole (**3c′**)

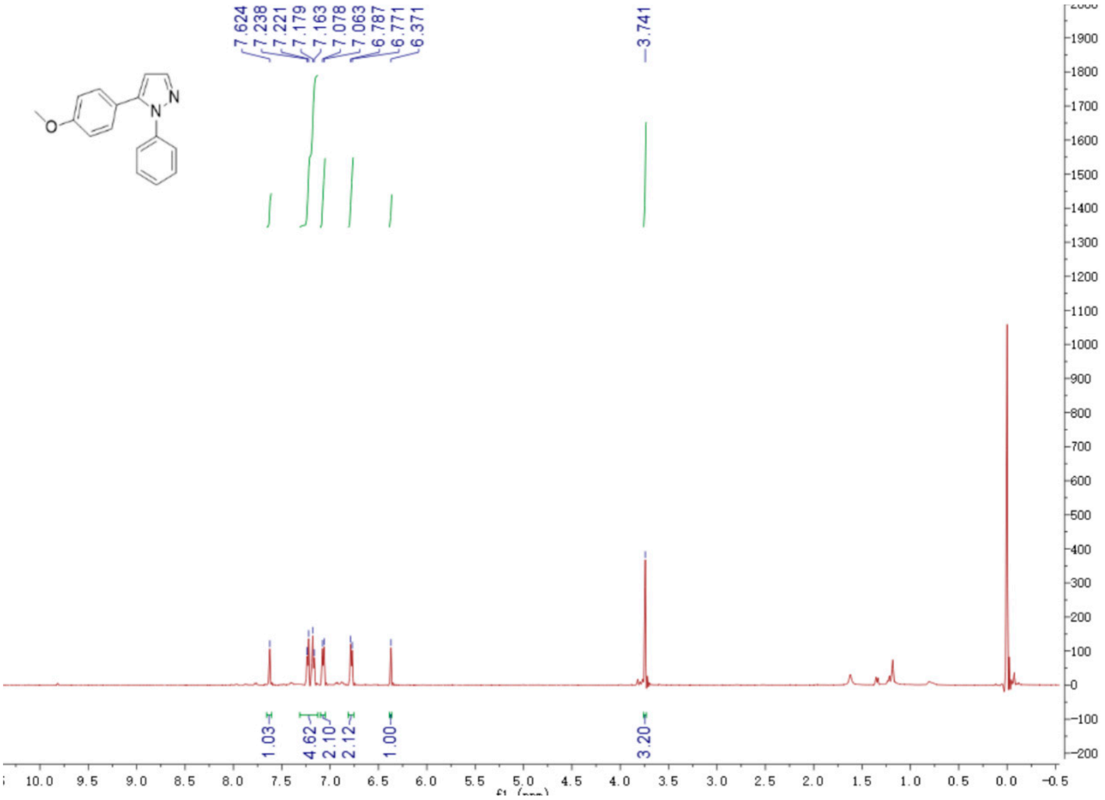

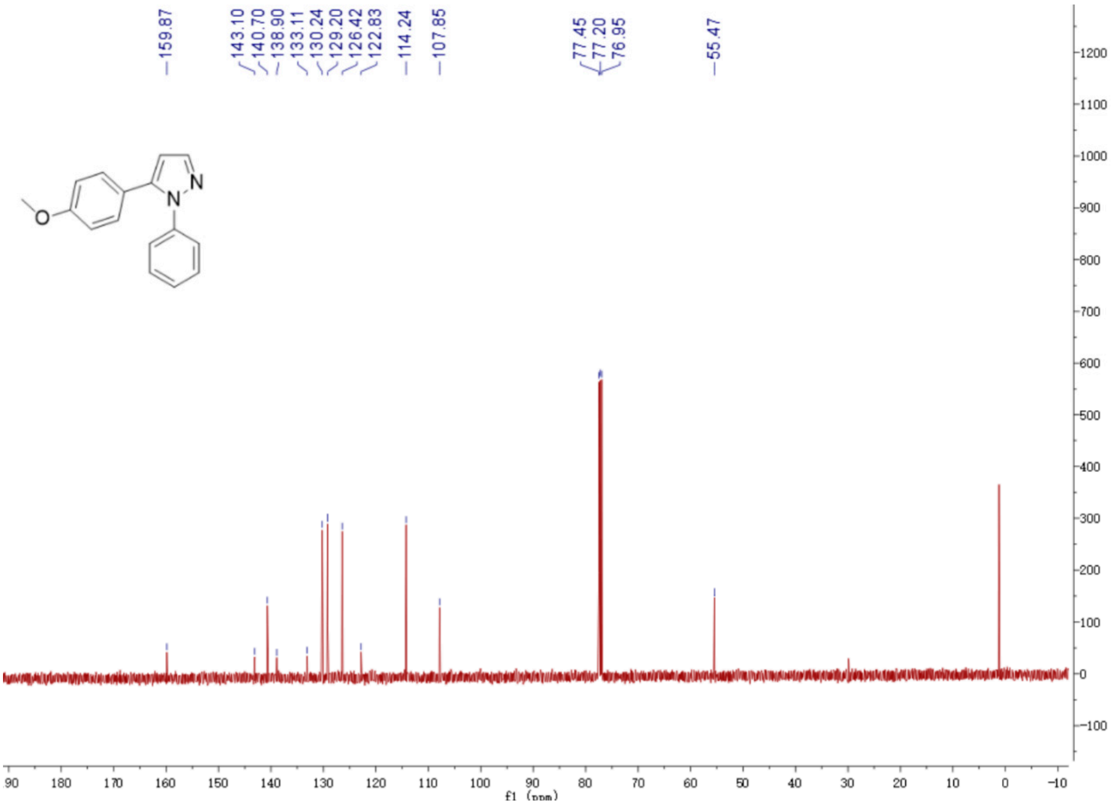

4-chloro-3-methyl-1-phenyl-5-(p-tolyl)-1H-pyrazole (**3d**)

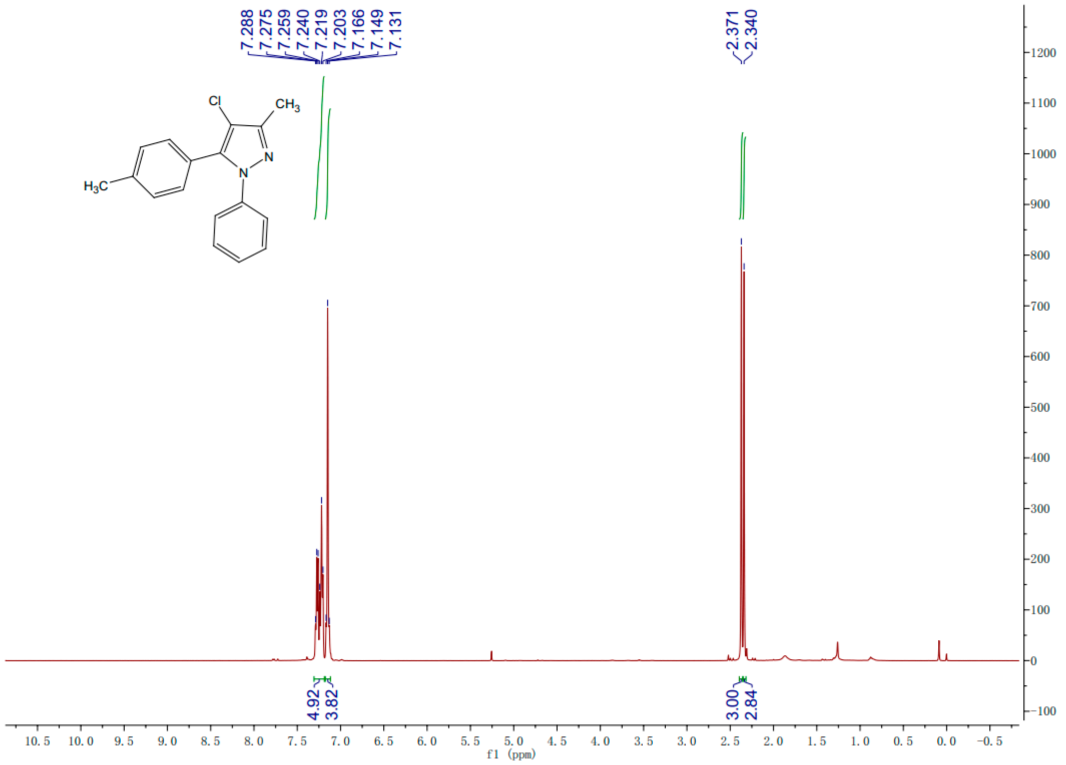

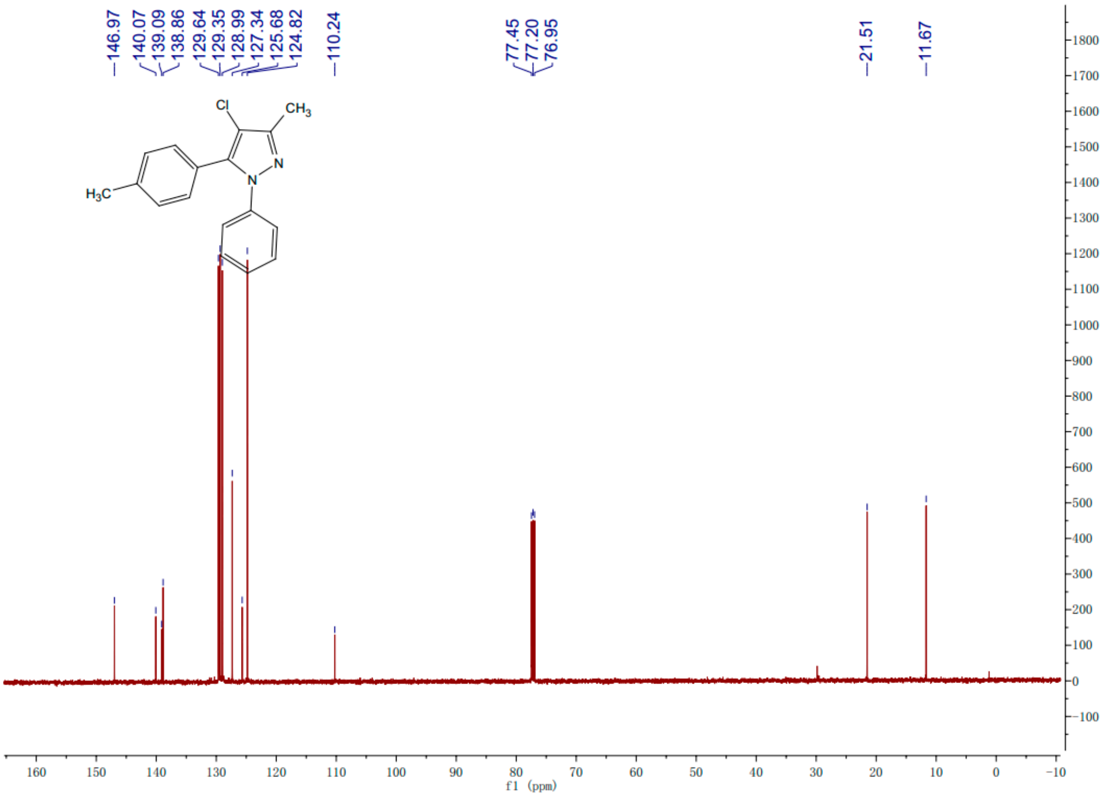

4-chloro-5-(4-methoxyphenyl)-3-methyl-1-phenyl-1H-pyrazole (**3e**)

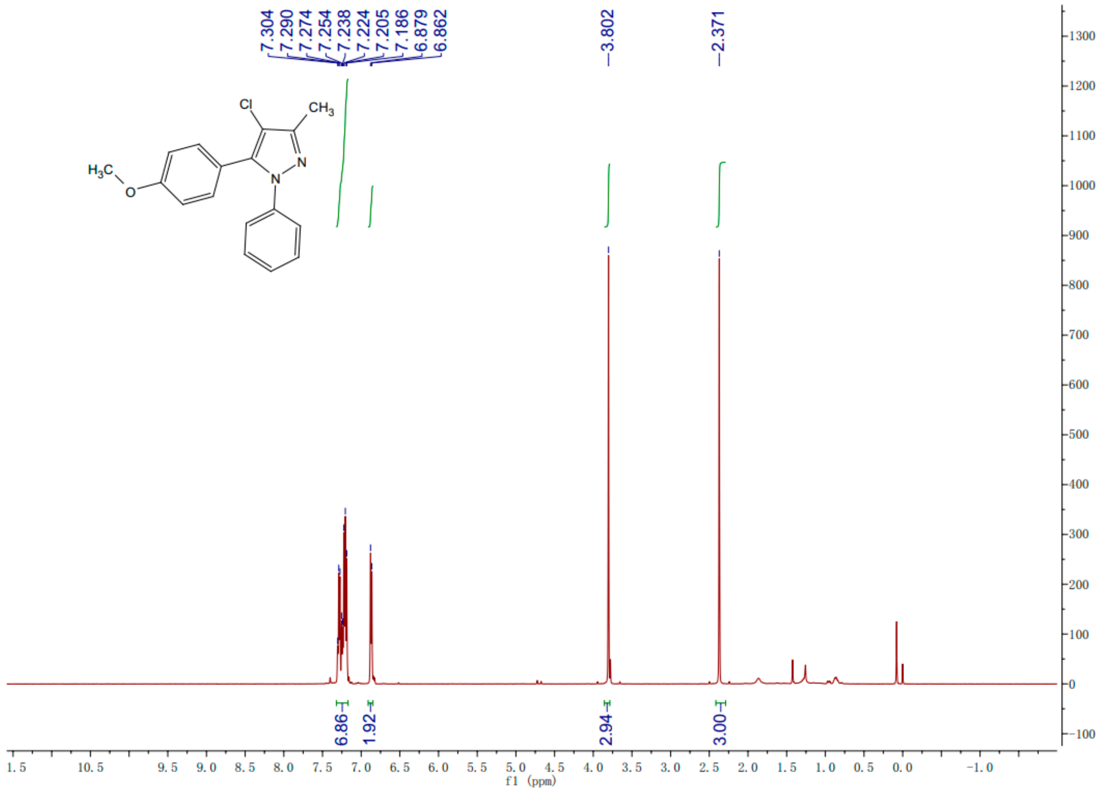

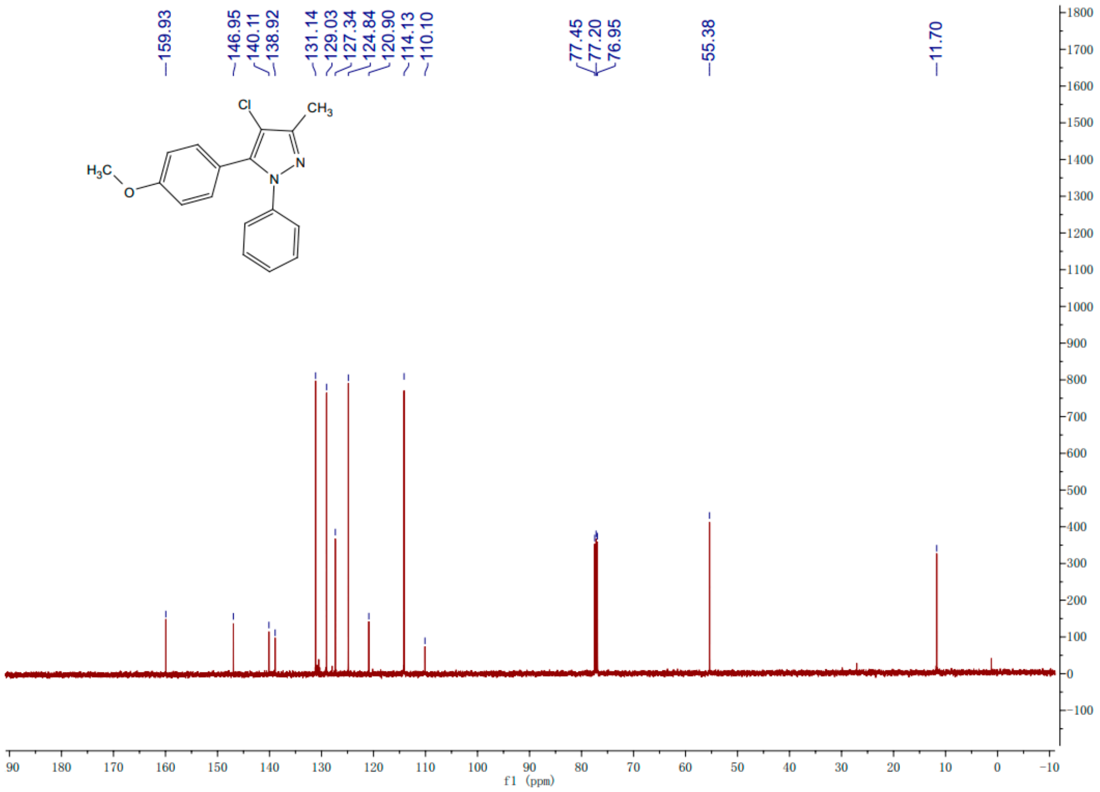

4-chloro-5-(4-fluorophenyl)-3-methyl-1-phenyl-1H-pyrazole (**3f**)

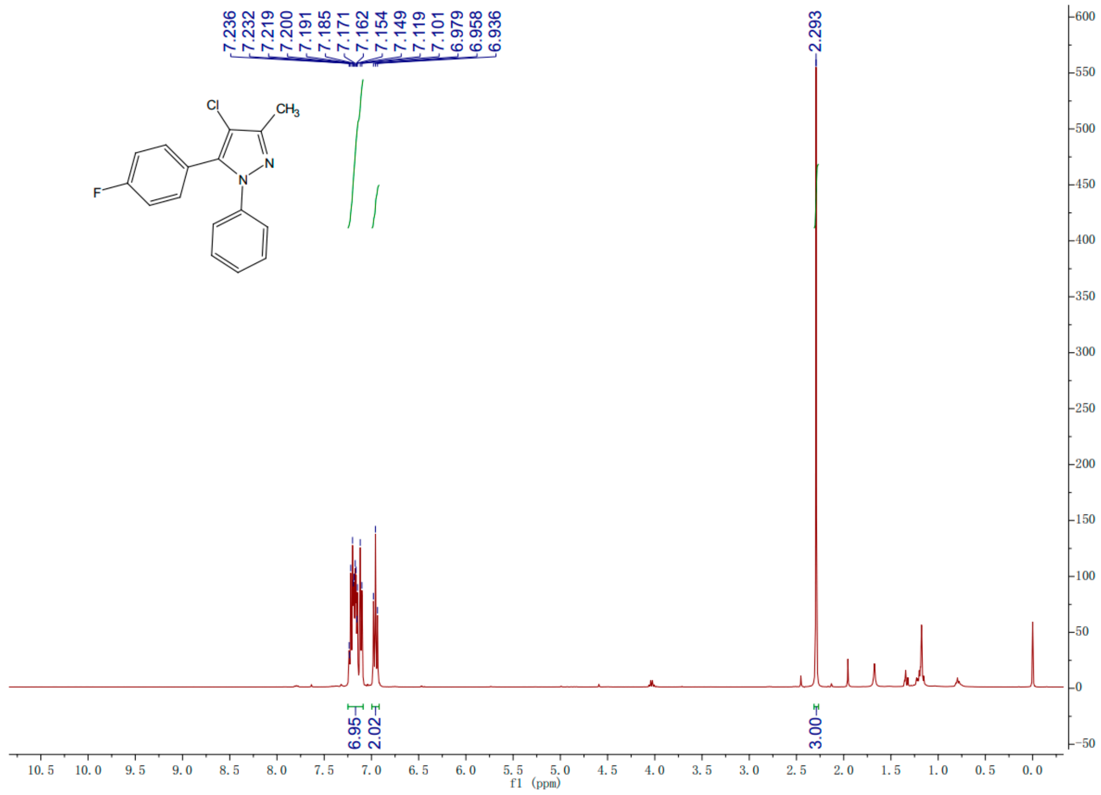

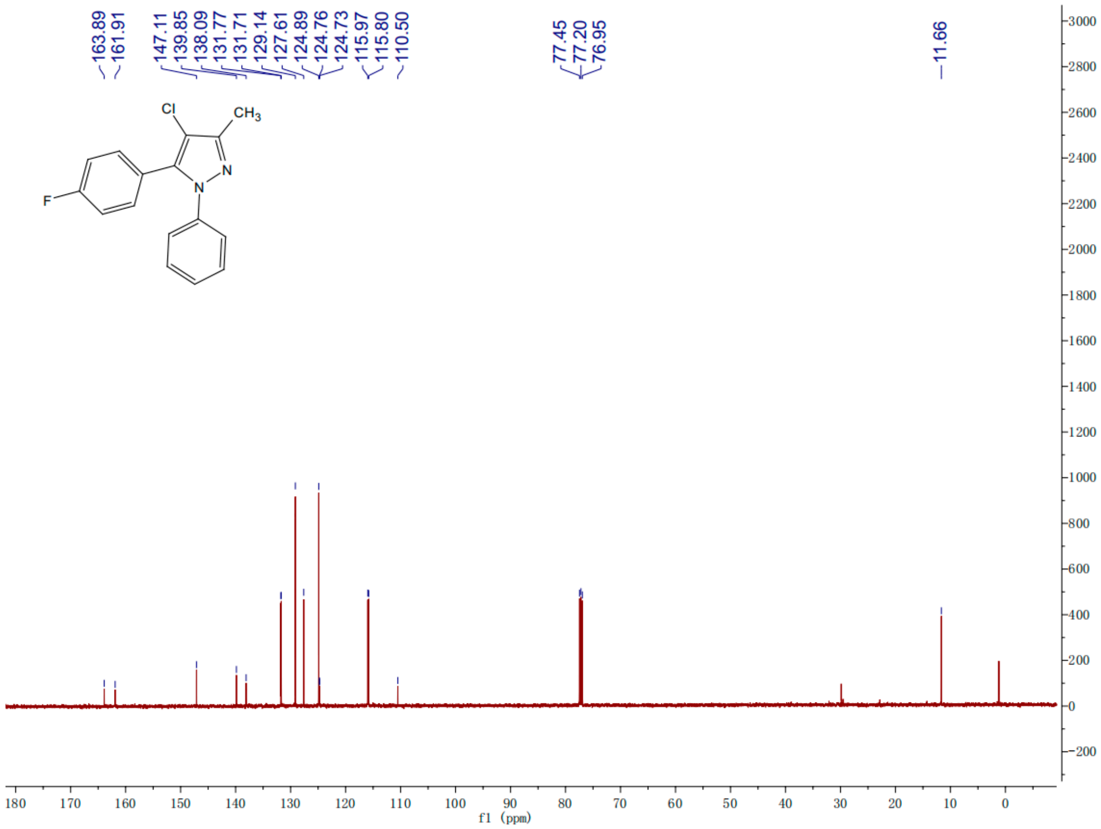

4-chloro-5-(4-chlorophenyl)-3-methyl-1-phenyl-1H-pyrazole (**3g**)

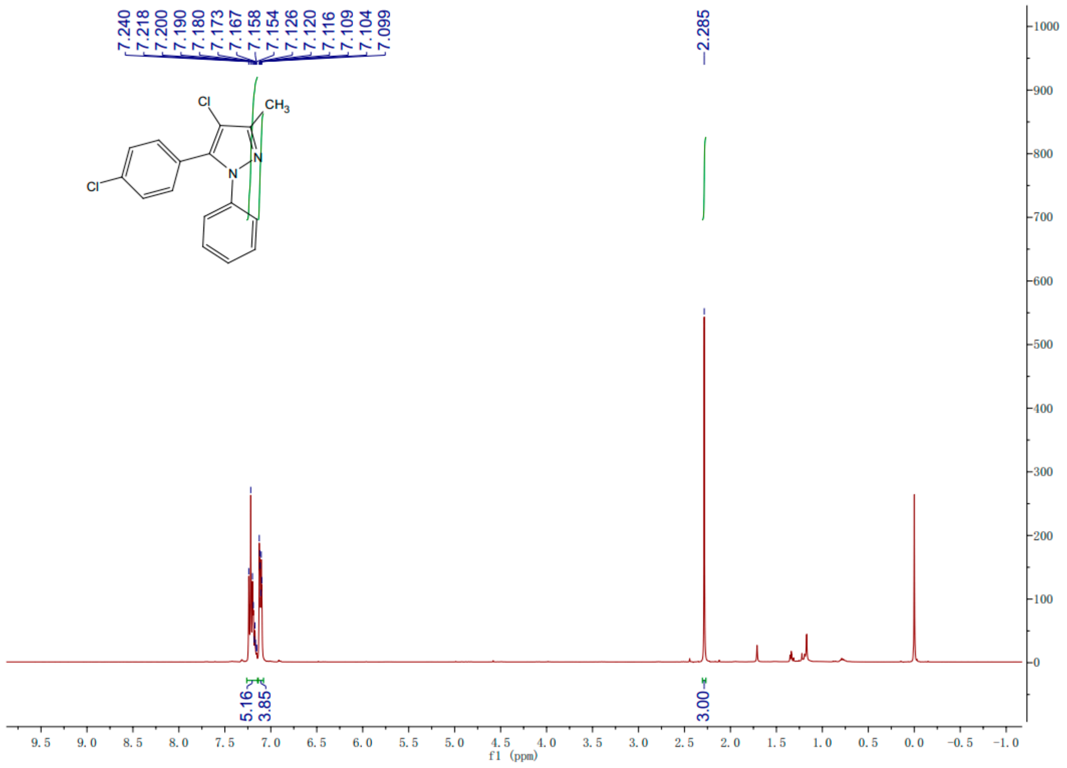

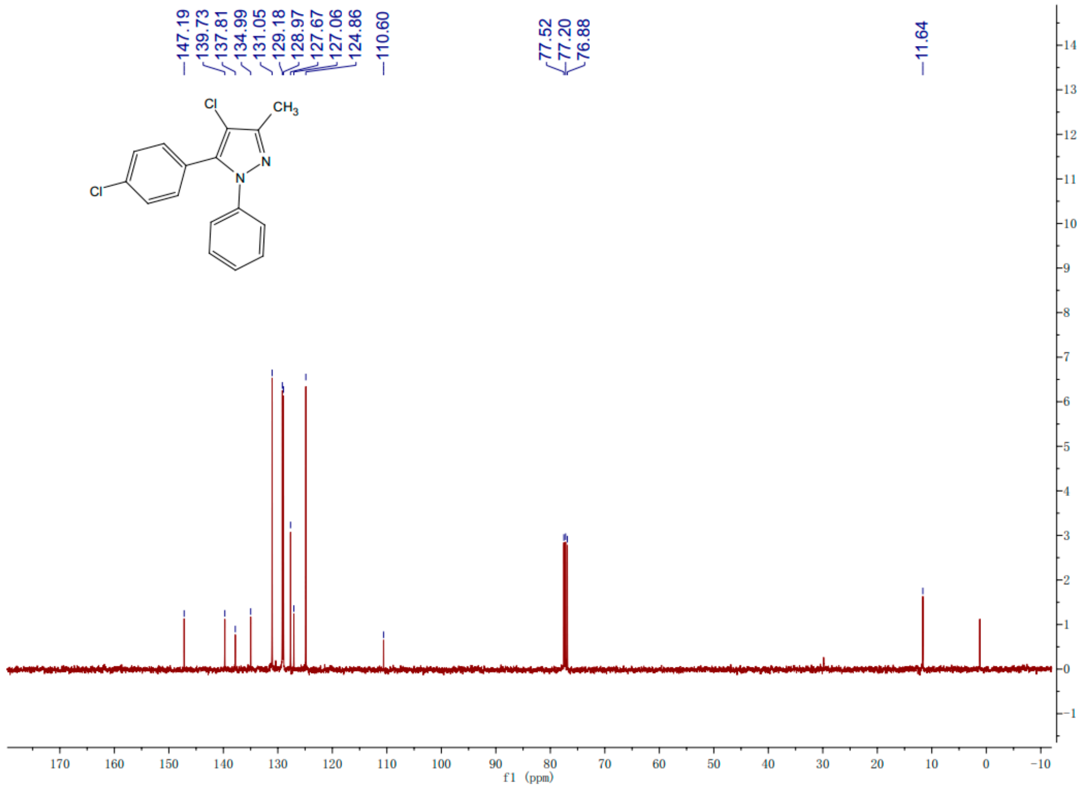

5-(4-bromophenyl)-4-chloro-3-methyl-1-phenyl-1H-pyrazole (**3h**)

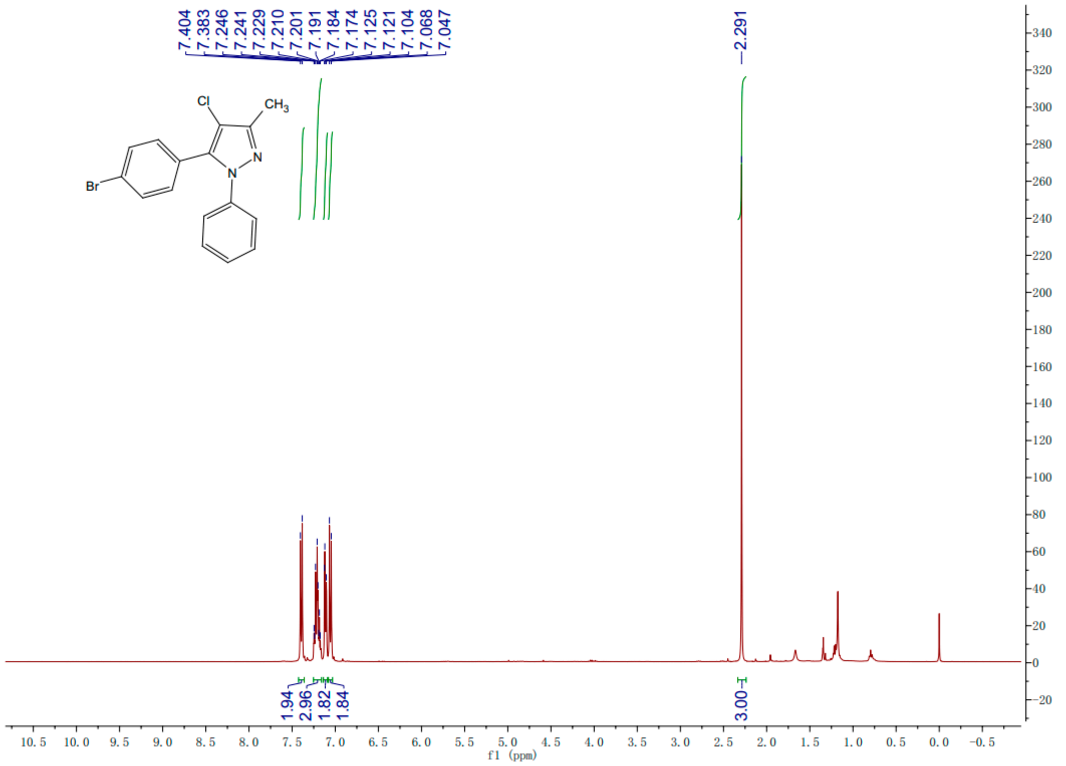

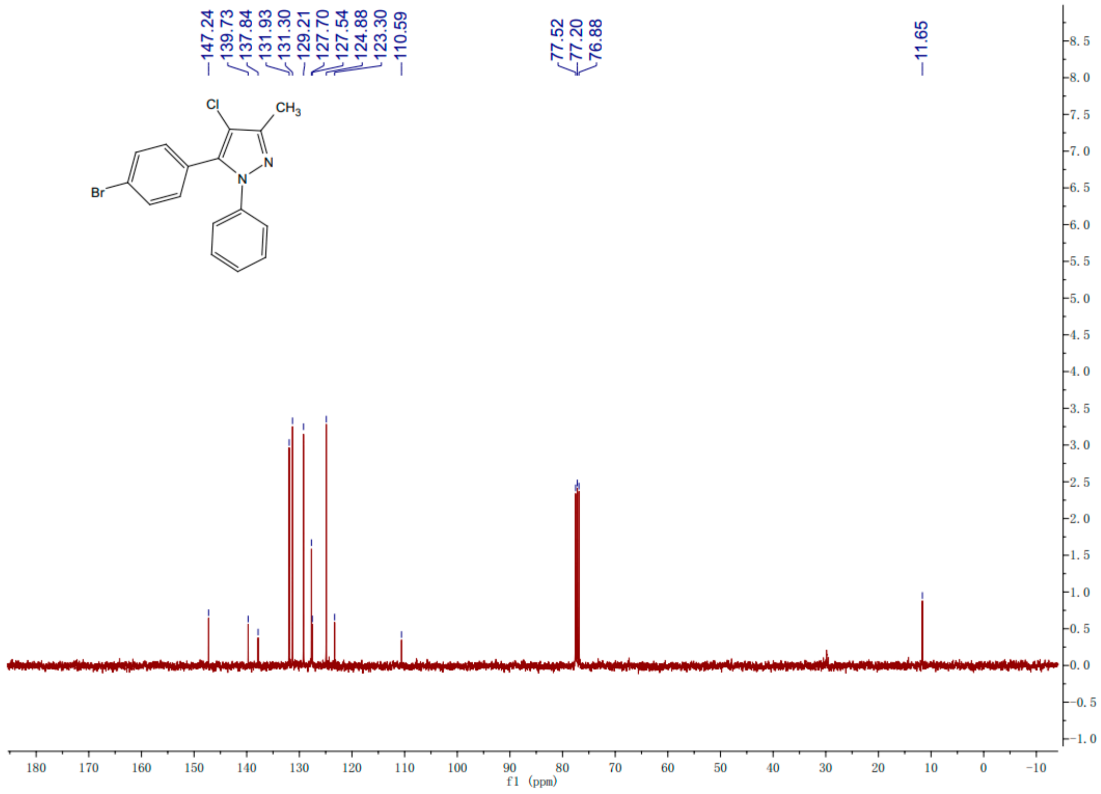

4-(4-chloro-3-methyl-1-phenyl-1H-pyrazol-5-yl)benzonitrile (**3i**)

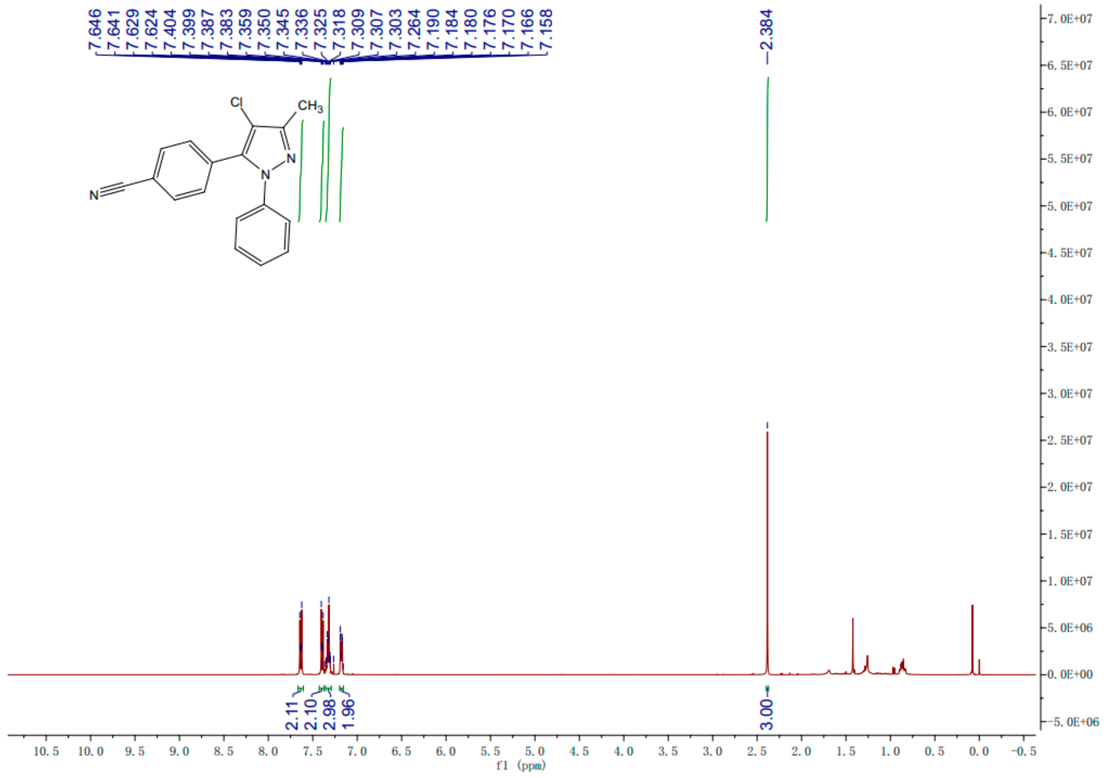

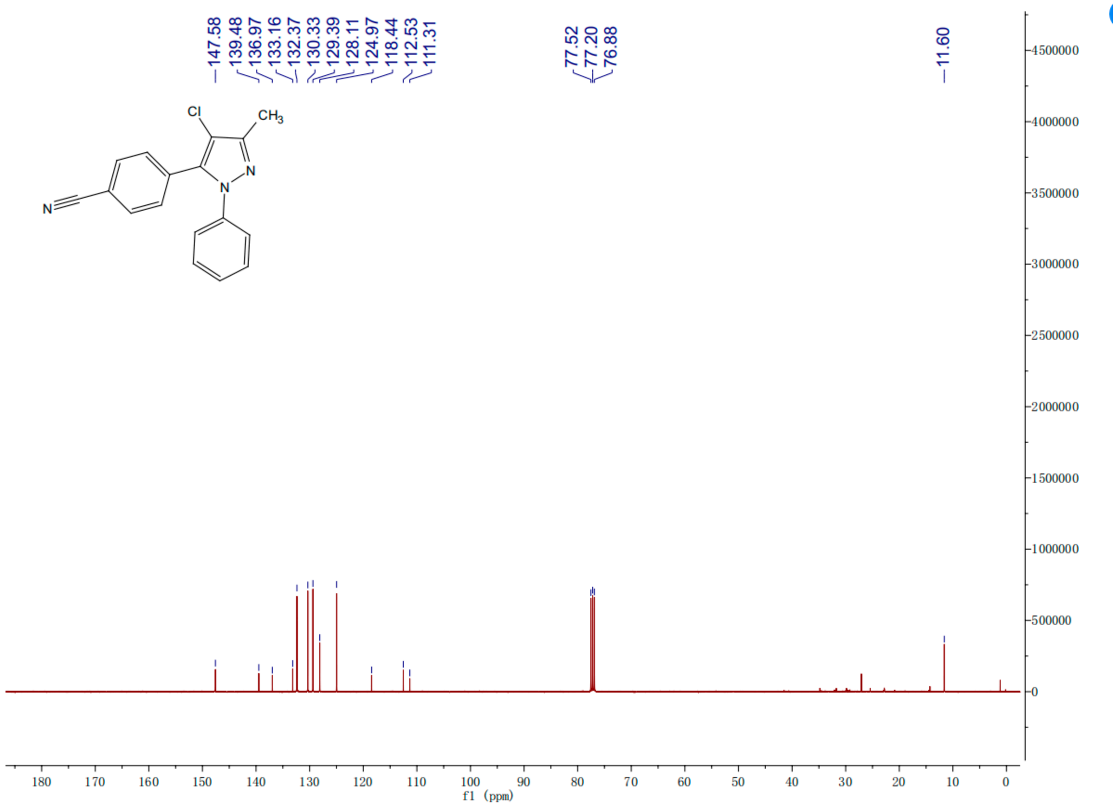

4-chloro-5-(3,4-dimethoxyphenyl)-3-methyl-1-phenyl-1H-pyrazole (**3j**)

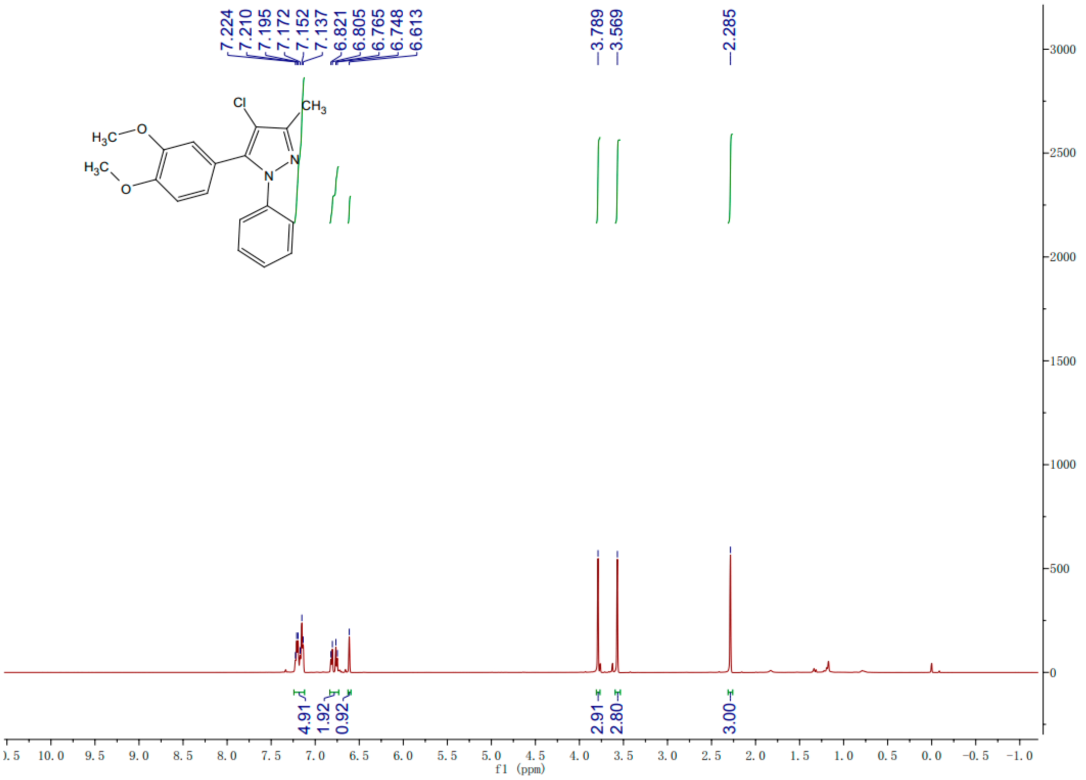

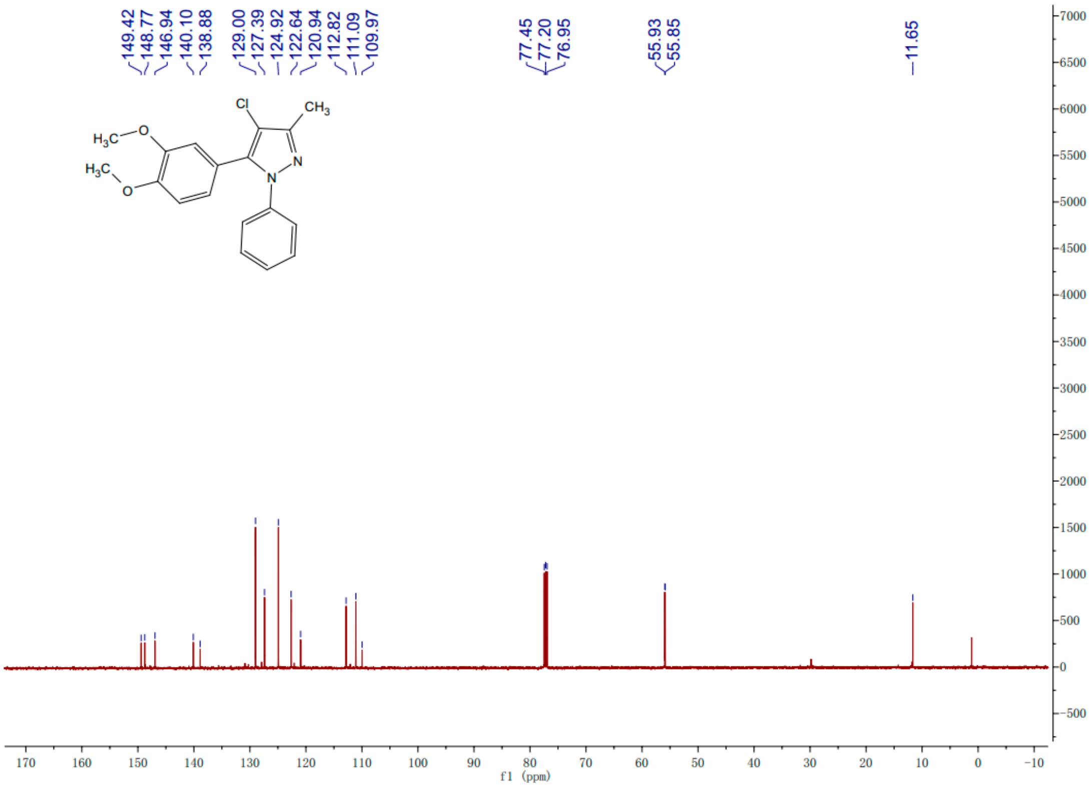

4-chloro-1,3-diphenyl-5-(p-tolyl)-1H-pyrazole (**3k**)

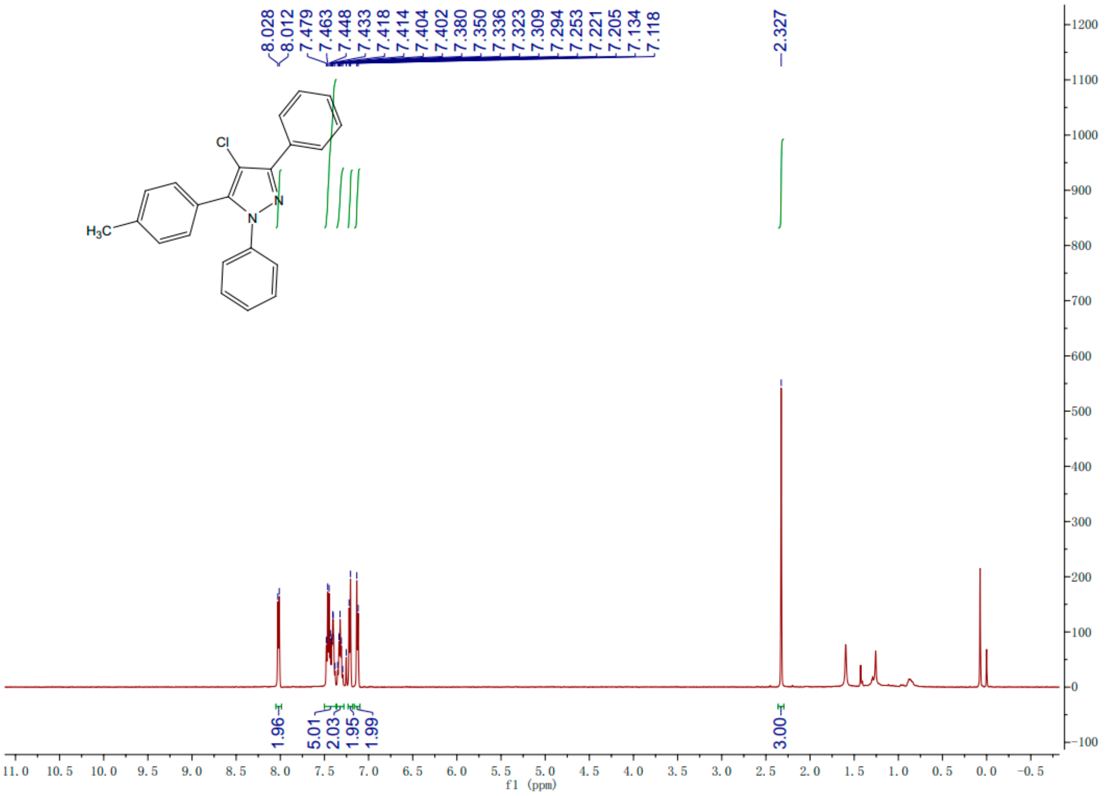

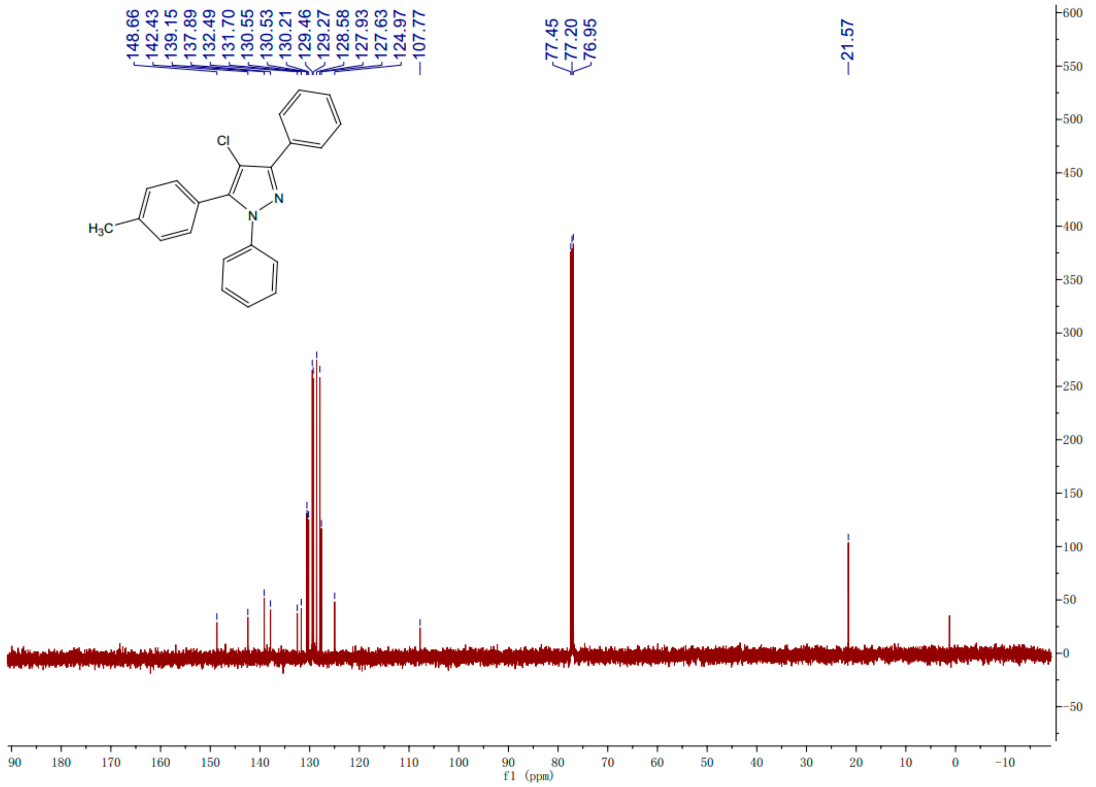

4-chloro-5-(4-methoxyphenyl)-1,3-diphenyl-1H-pyrazole (**3l**)

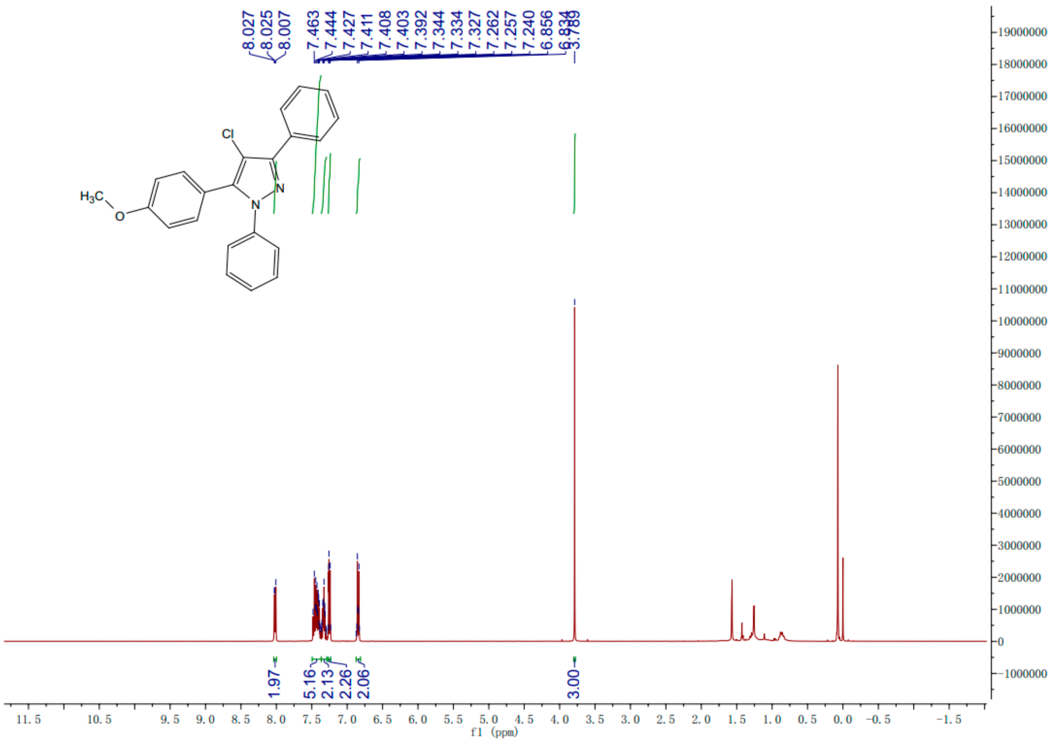

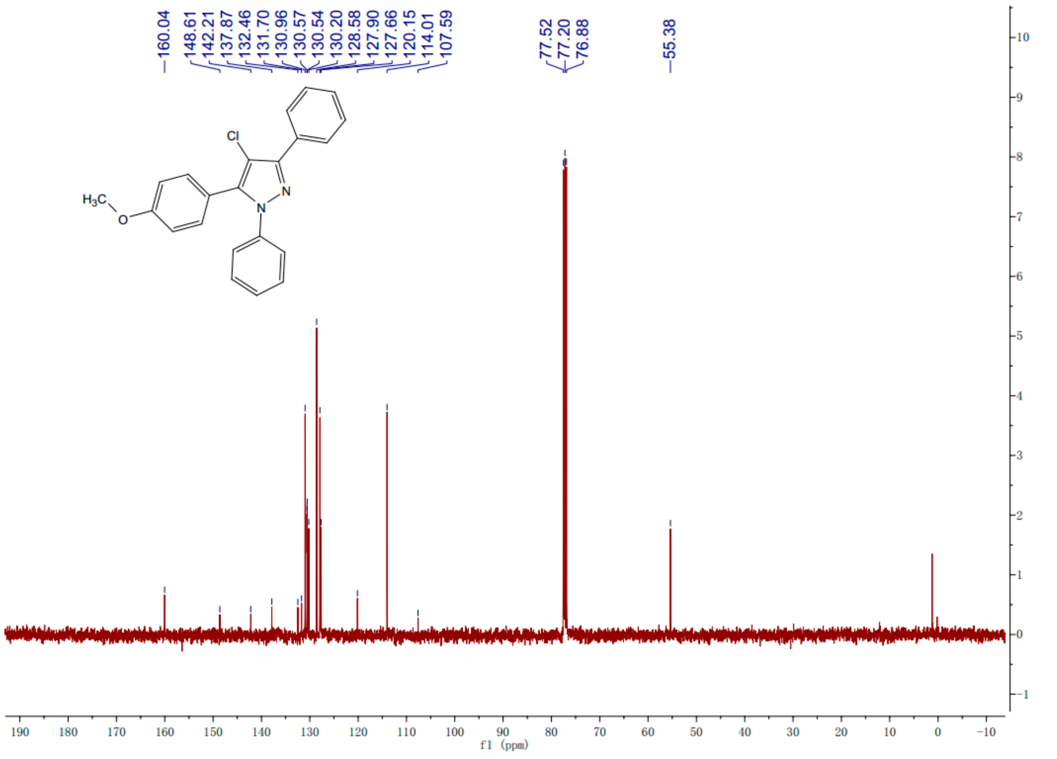

4-chloro-5-(4-chlorophenyl)-1,3-diphenyl-1H-pyrazole (**3m**)

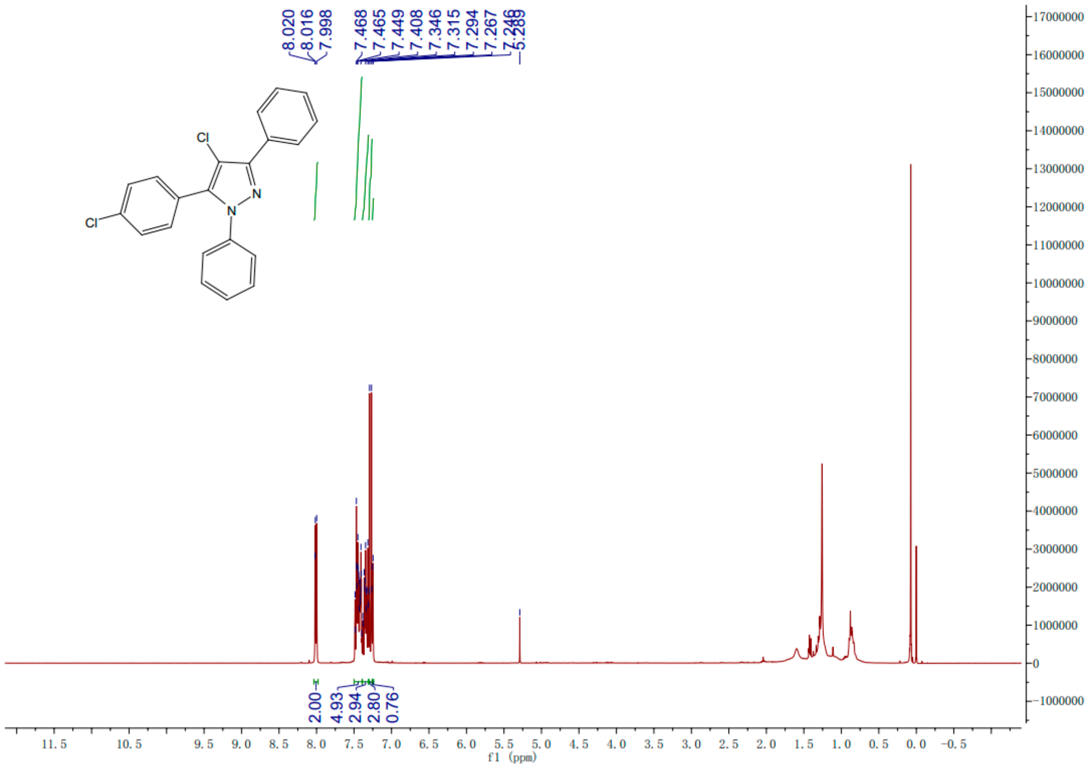

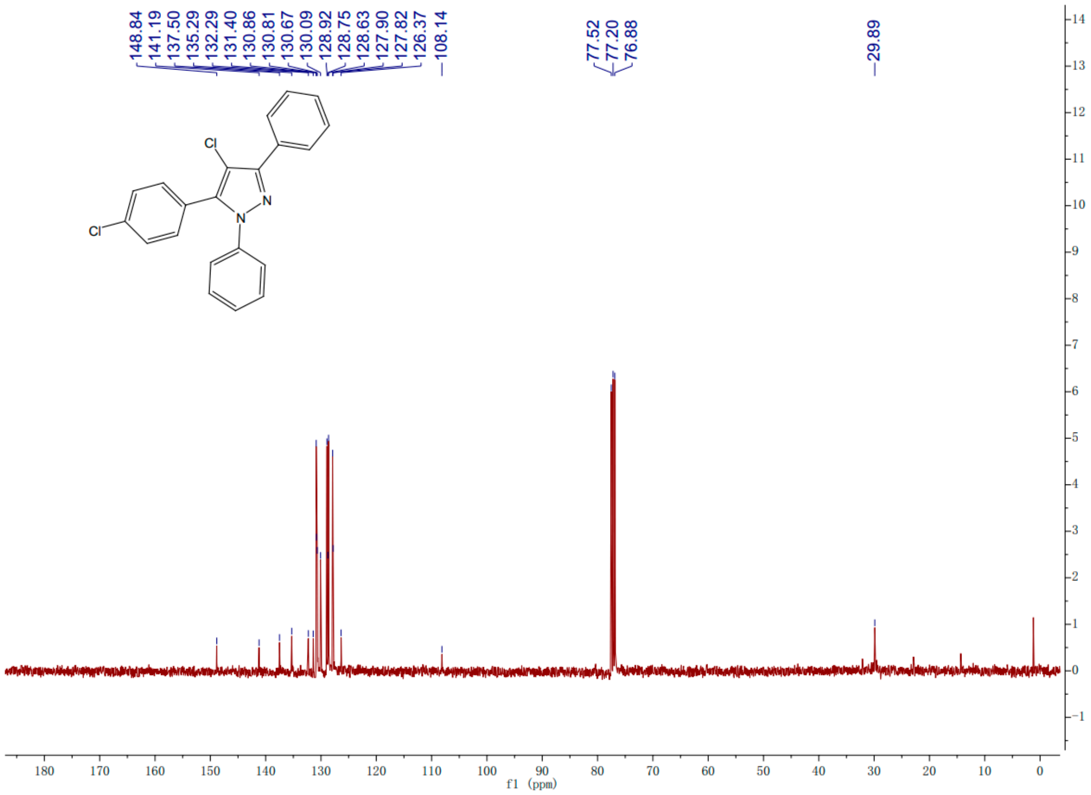

5-(4-bromophenyl)-4-chloro-1,3-diphenyl-1H-pyrazole (**3n**)

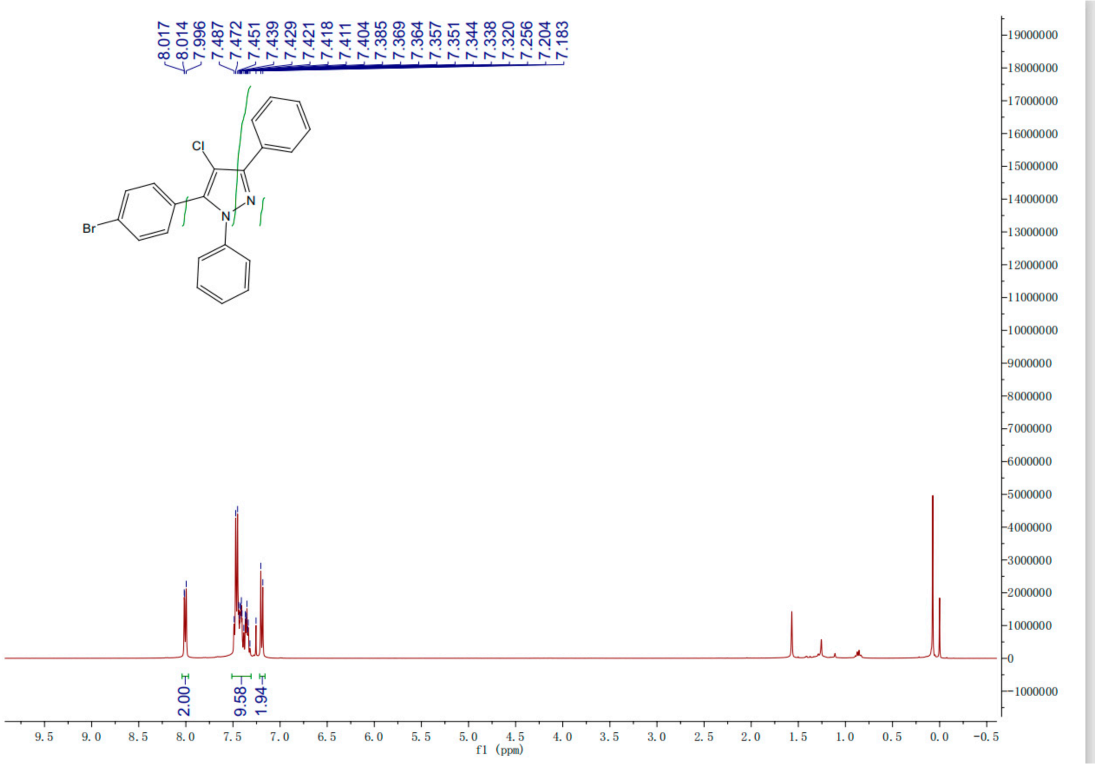

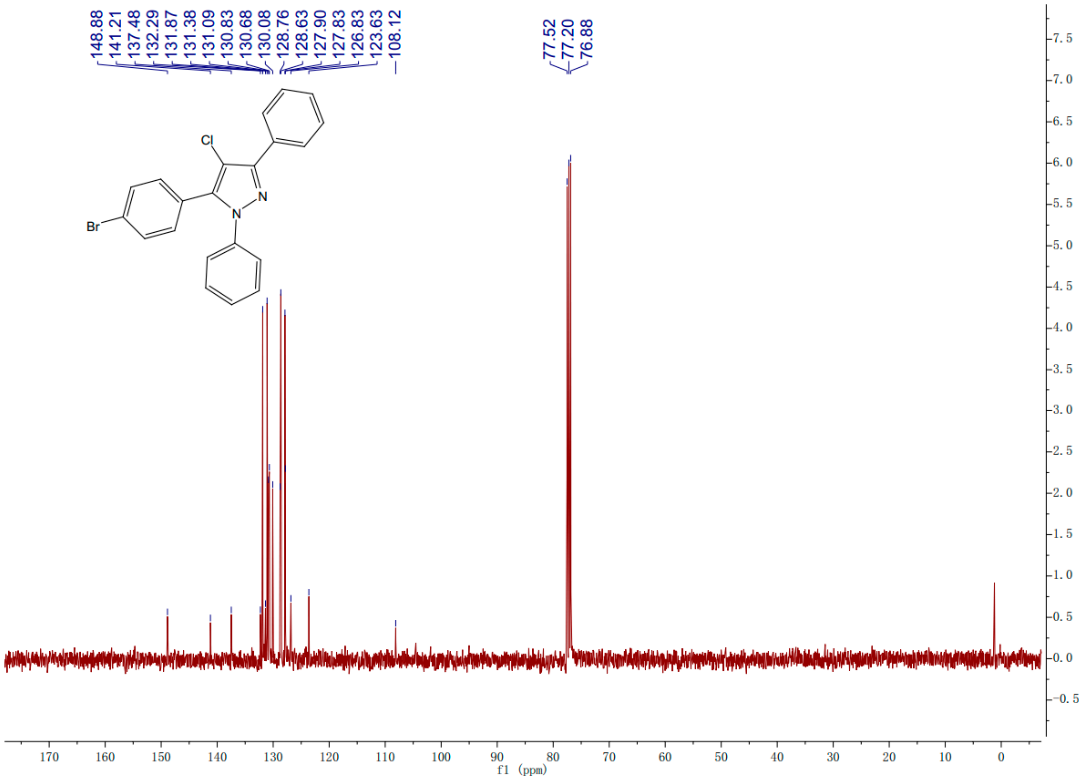

4-chloro-1-phenyl-3,5-di-p-tolyl-1H-pyrazole (**3o**)

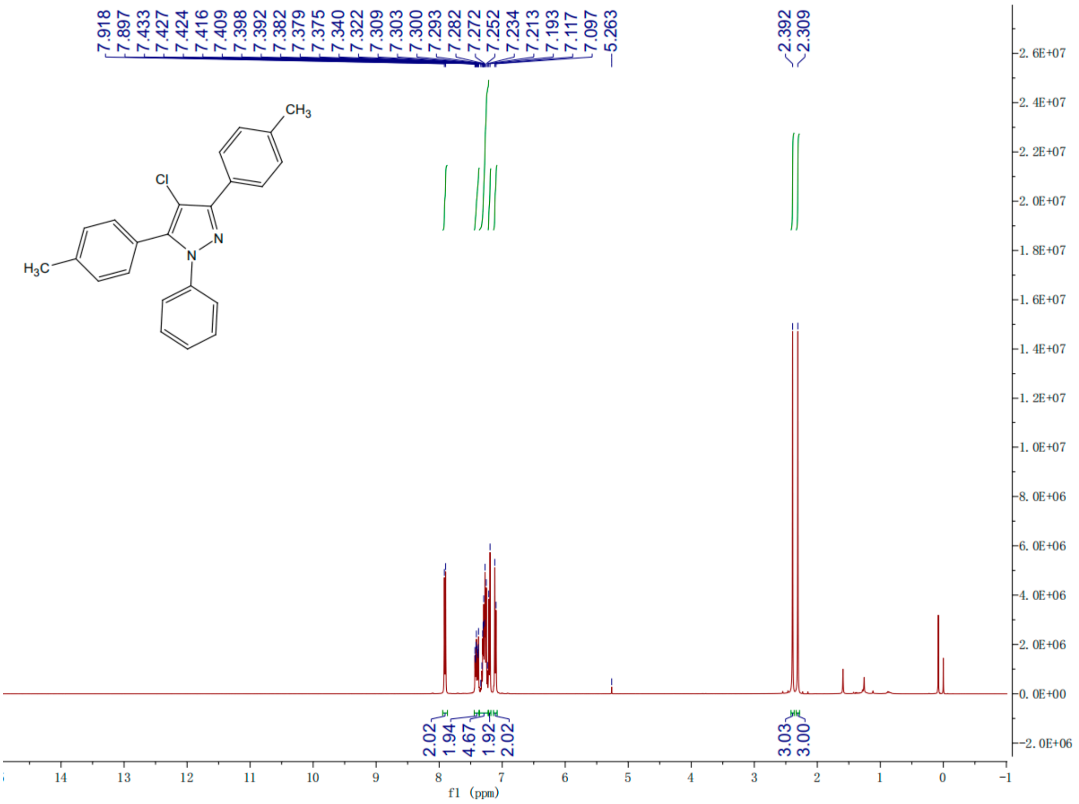

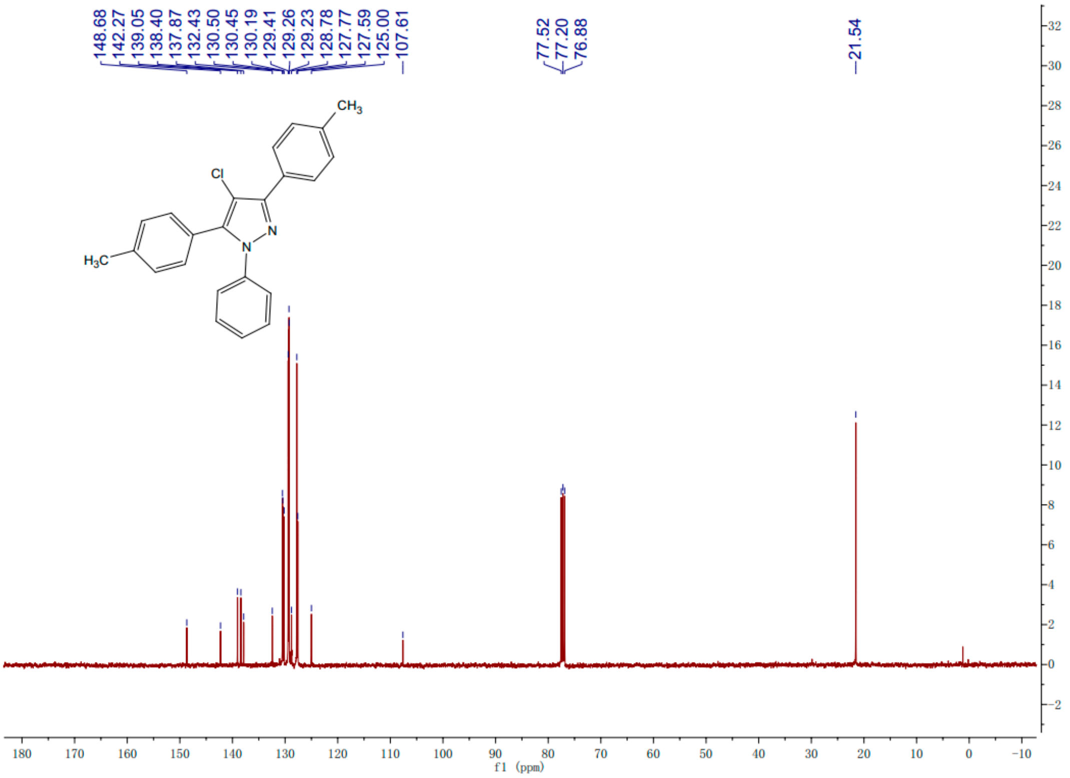

4-methyl-1,5-diphenyl-1H-pyrazole(**3p'**)

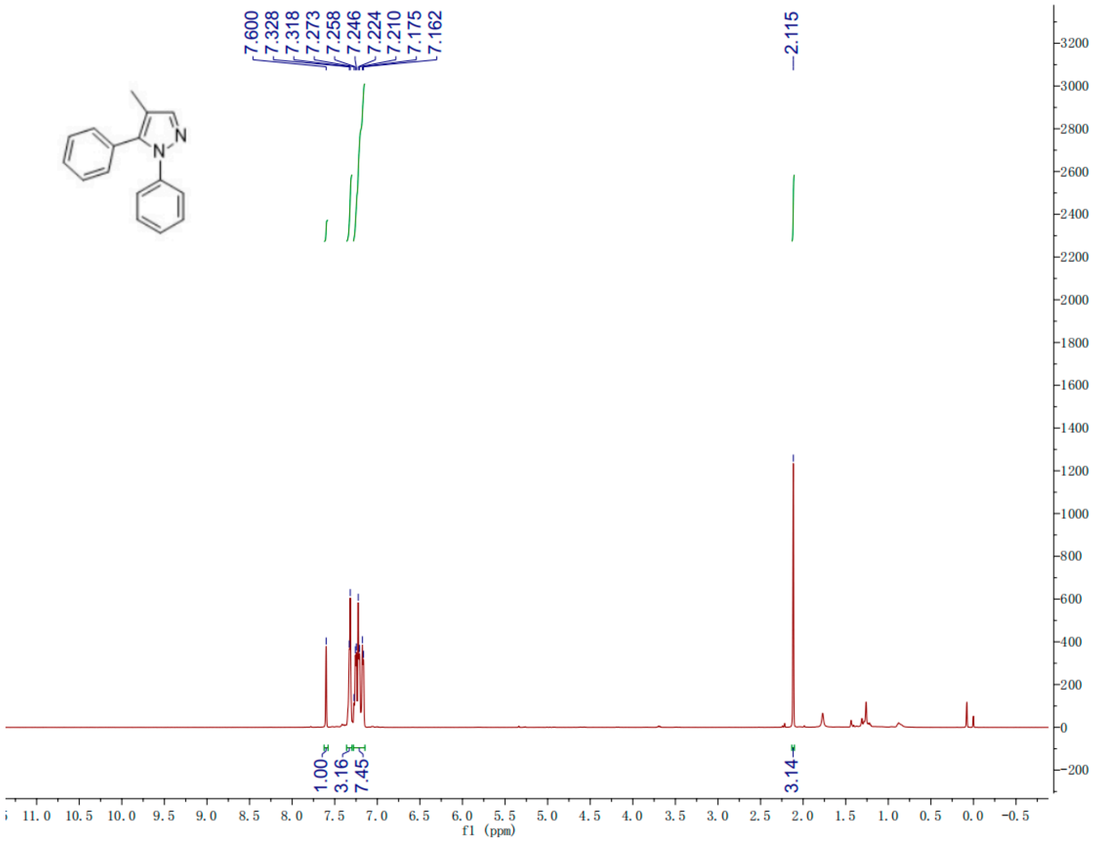

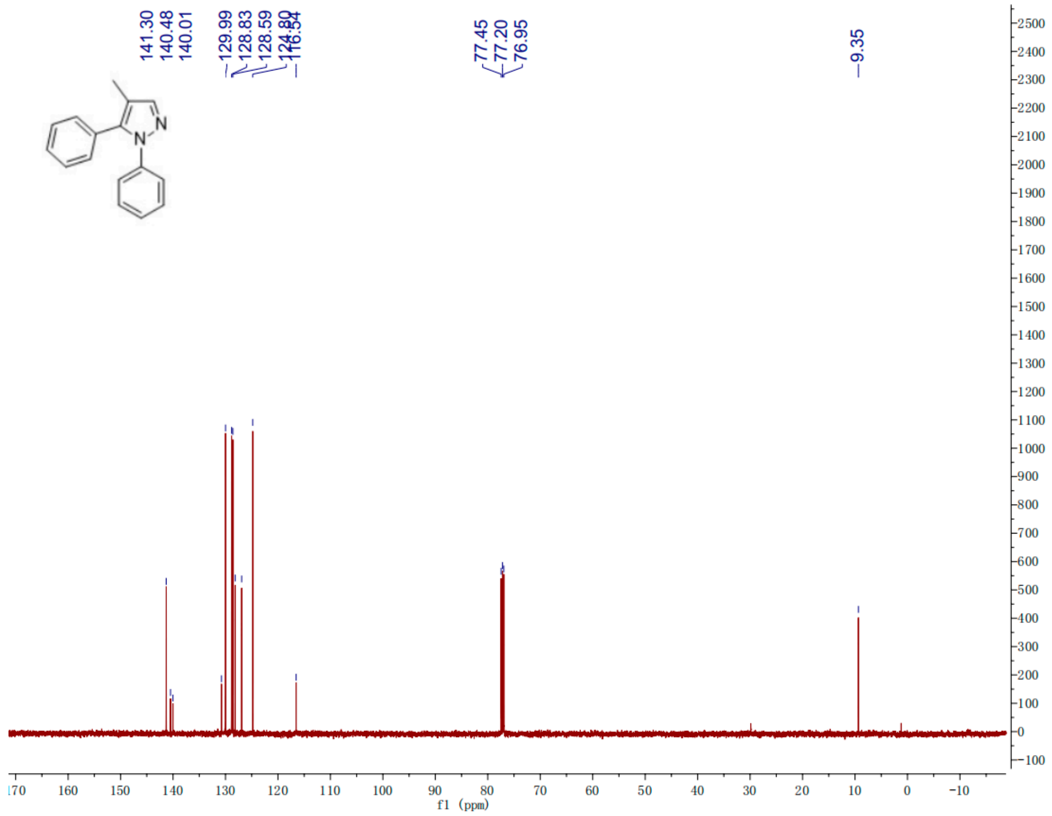

3-methyl-1,4,5-triphenyl-1H-pyrazole (**4a**)

^{1}H NMR (400 MHz, $CDCl_3$)

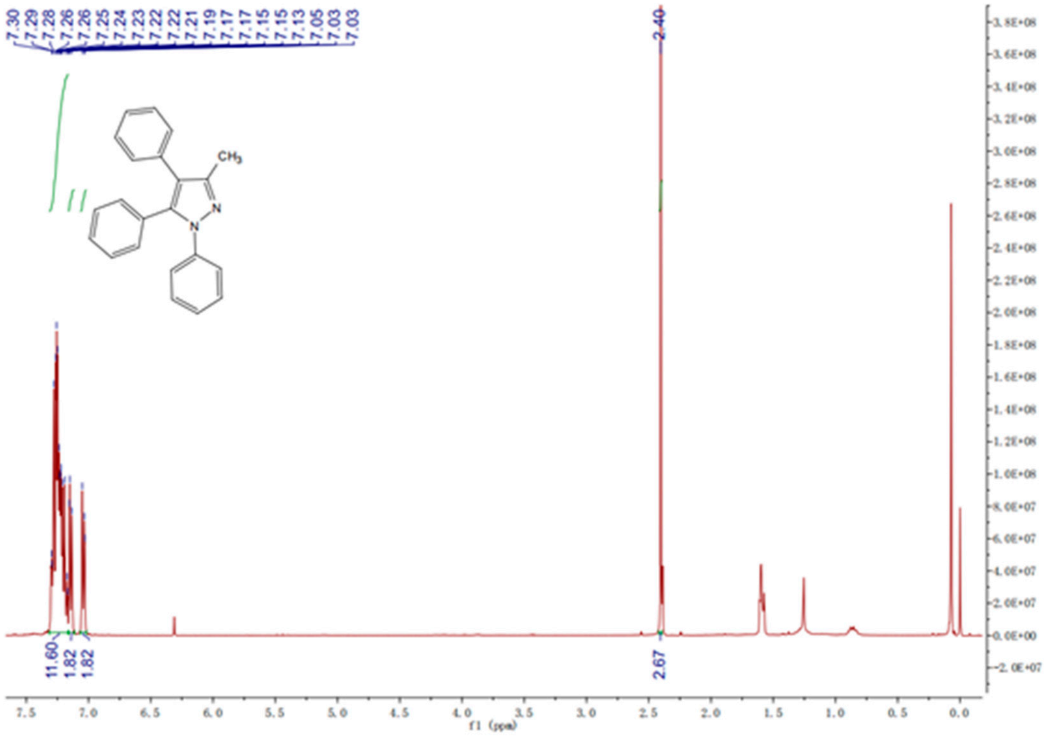

^{13}C {^{1}H} NMR (100 MHz, $CDCl_3$)

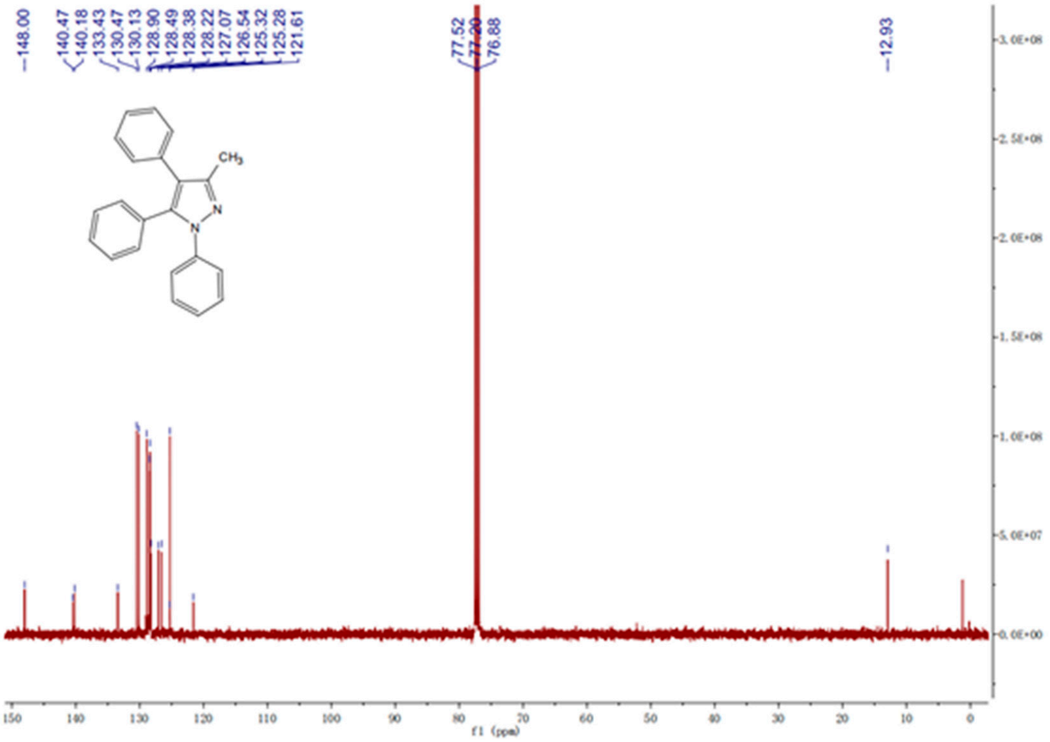

3-methyl-1,5-diphenyl-4-(p-tolyl)-1H-pyrazole (**4b**)

^{1}H NMR (400 MHz, $CDCl_3$)

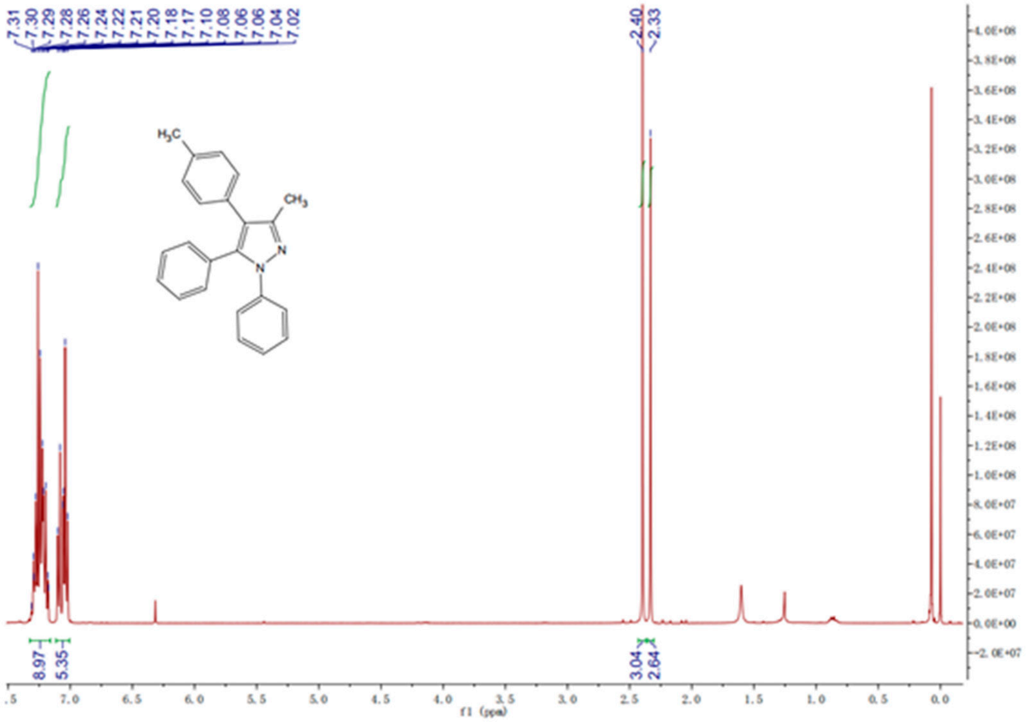

^{13}C {^{1}H} NMR (100 MHz, $CDCl_3$)

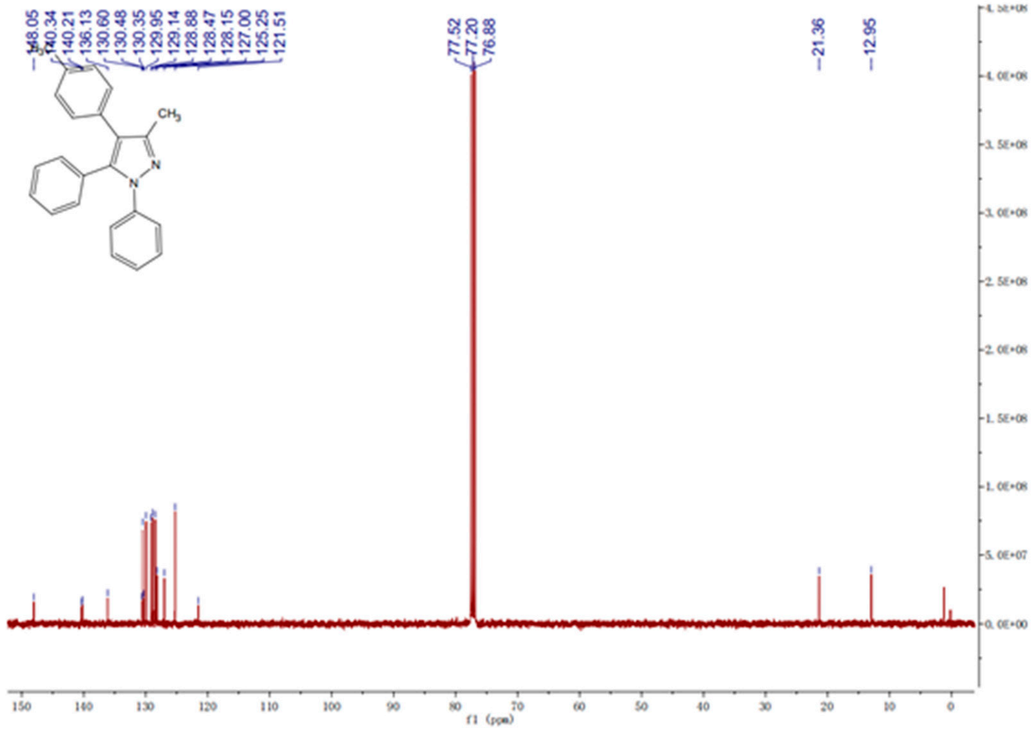

4-(4-methoxyphenyl)-3-methyl-1,5-diphenyl-1H-pyrazole (**4c**)

^{1}H NMR (400 MHz, $CDCl_3$)

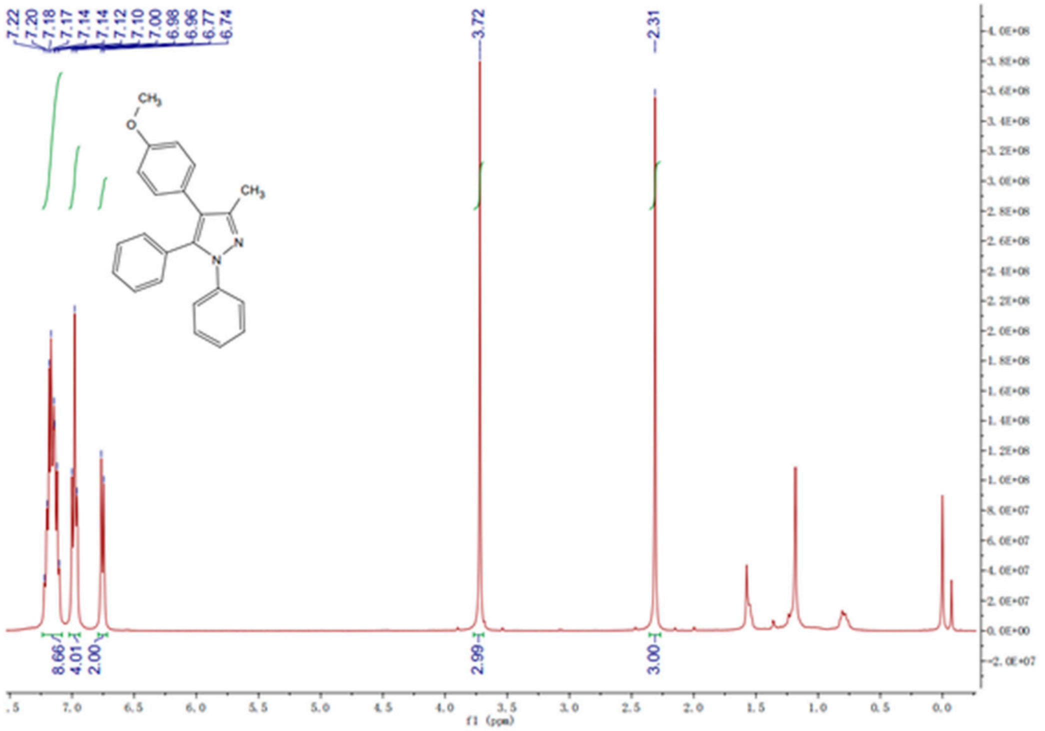

^{13}C {^{1}H} NMR (100 MHz, $CDCl_3$)

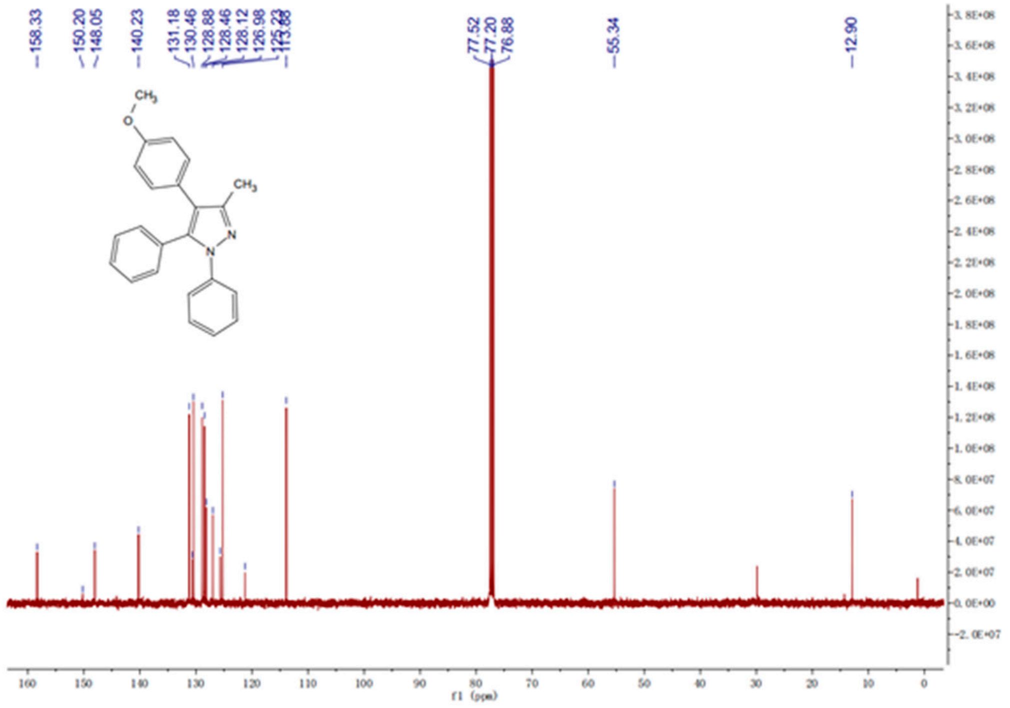

3-methyl-1,5-diphenyl-4-(4-(trimethylsilyl)phenyl)-1H-pyrazole (**4d**)

^{1}H NMR (400 MHz, $CDCl_3$)

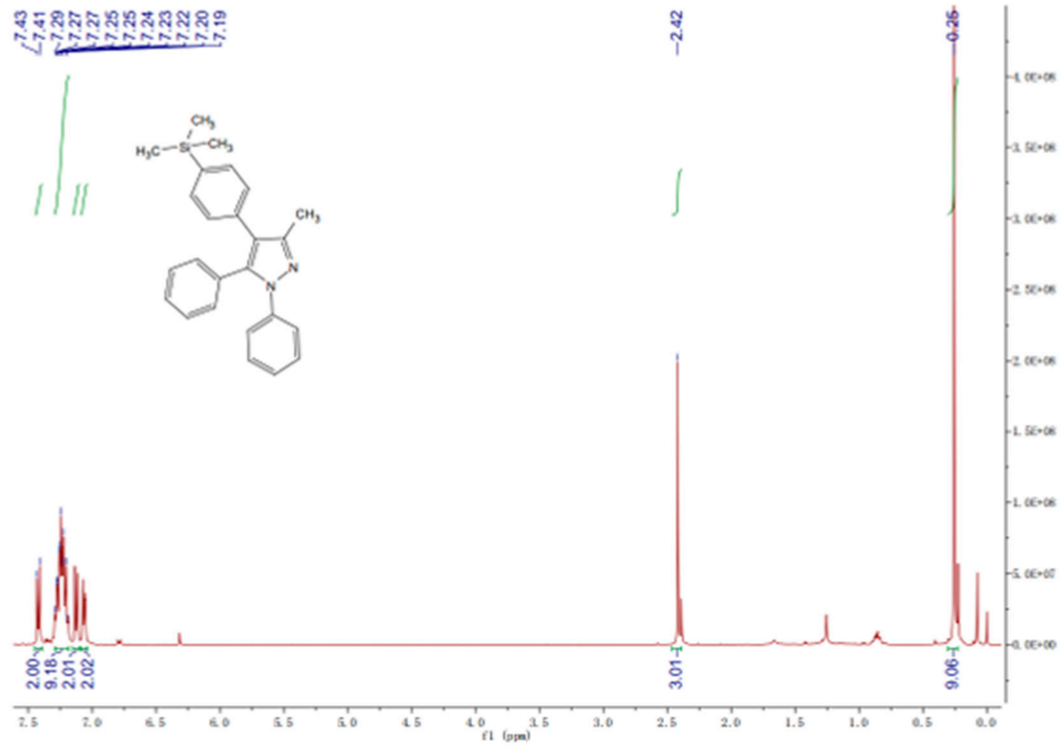

^{13}C {^{1}H} NMR (100 MHz, $CDCl_3$)

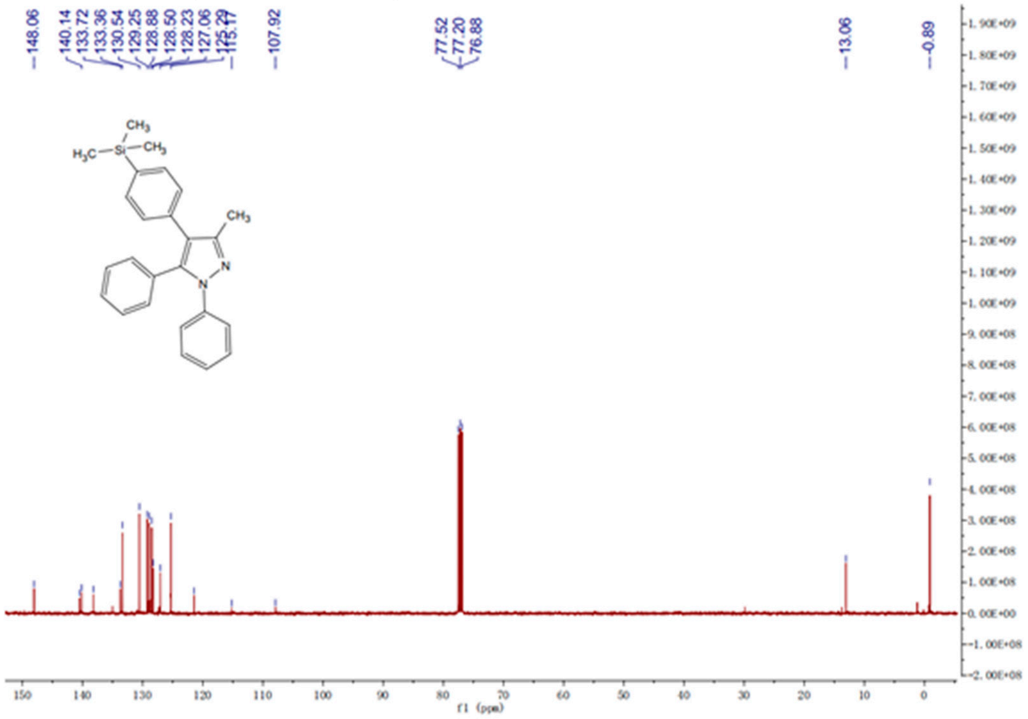

4-(4-chlorophenyl)-3-methyl-1,5-diphenyl-1H-pyrazole (**4e**)

^{1}H NMR (400 MHz, $CDCl_3$)

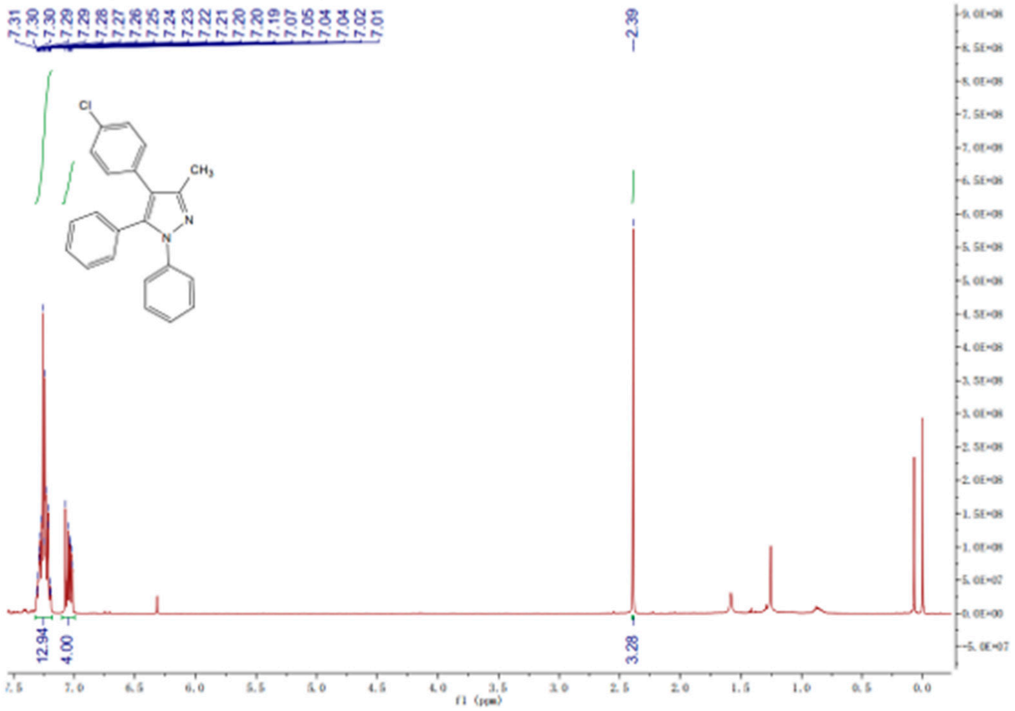

^{13}C {^{1}H} NMR (100 MHz, $CDCl_3$)

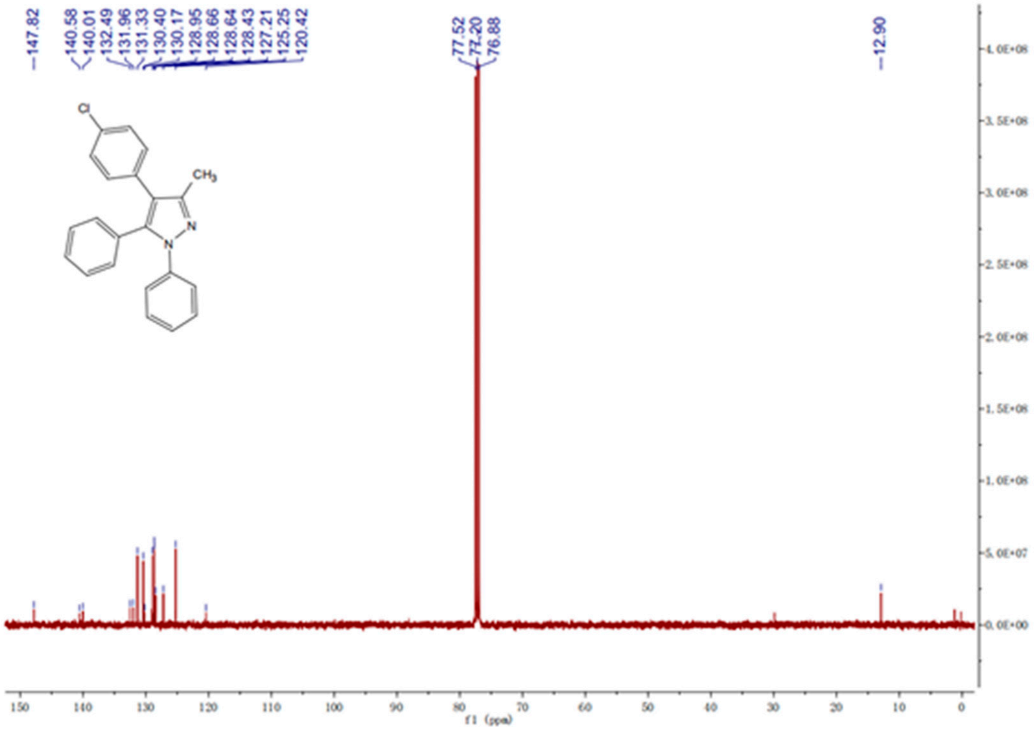

4-([1,1'-biphenyl]-4-yl)-3-methyl-1,5-diphenyl-1H-pyrazole (**4f**)

^{1}H NMR (400 MHz, $CDCl_3$)

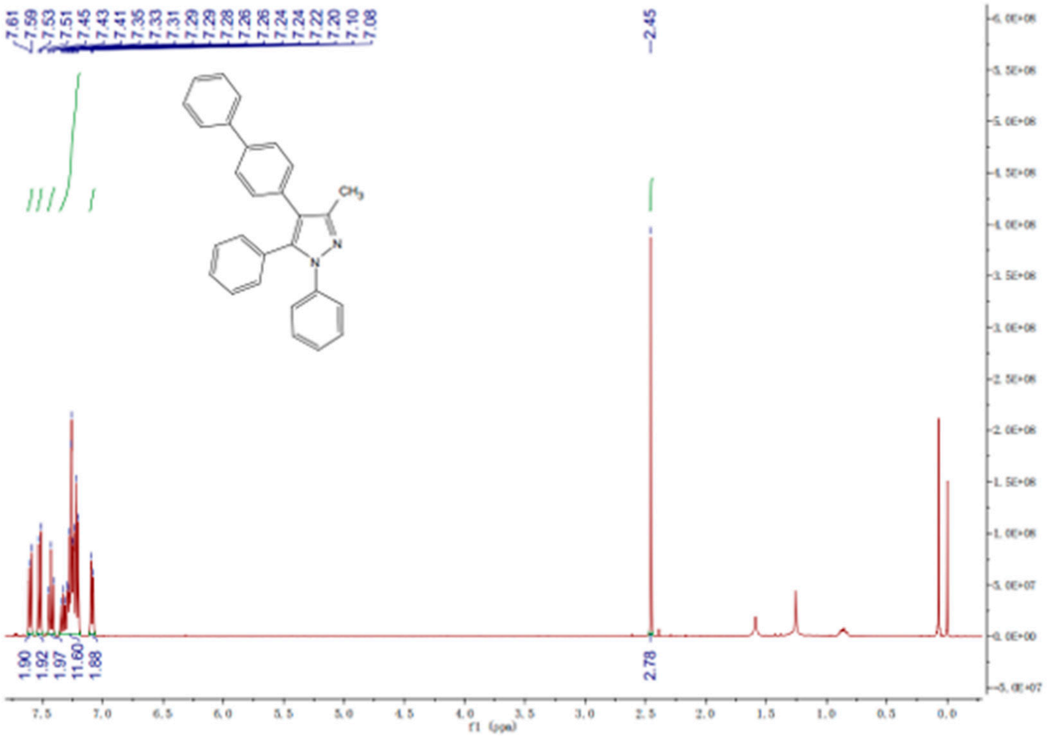

^{13}C {^{1}H} NMR (100 MHz, $CDCl_3$)

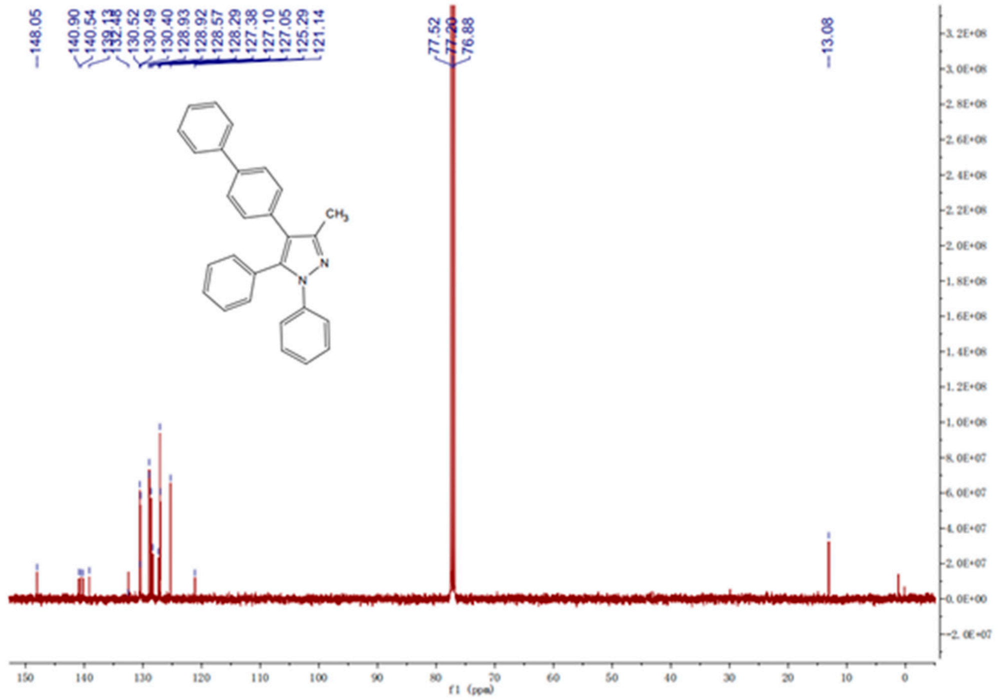

References

[1] Liu, J.; Xu, E.; Jiang, J.; Huang, Z.; Zheng, L.; Liu, Z.-Q. Copper-mediated tandem ring-opening/cyclization reactions of cyclopropanols with aryldiazonium salts: synthesis of N-arylpyrazoles. Chemical Communication. 2020, 56, 2202-2205. DOI: https://doi. org/10.1039/c9cc09657d.

[2] Roy, S.; Chatterjee, R.; Kisan, P.; Dandela, R. Ultrasound-assisted synthesis of 1,5-disubstituted pyrazoles via HFIP-mediated cascade cyclization of enaminones with aryl hydrazine. Tetrahedron Letters. 2024, 149, 155277-155281. DOI: https://doi. org/10.1016/j.tetlet.2024.155277.

[3] Kashiwa, M.; Kuwata, Y.; Sonoda, M.; Tanimori S. Oxone-mediated facile access to substituted pyrazoles. Tetrahedron. 2016, 72, 304-311. DOI: 10.1002/chin.201620125.
